# GABA_**A**_ Receptor Coupling Junction and Pore *GABRB3* Mutations are Linked to Early-Onset Epileptic Encephalopathy

**DOI:** 10.1038/s41598-017-16010-3

**Published:** 2017-11-21

**Authors:** Ciria C. Hernandez, Yujia Zhang, Ningning Hu, Dingding Shen, Wangzhen Shen, Xiaoyan Liu, Weijing Kong, Yuwu Jiang, Robert L. Macdonald

**Affiliations:** 1 Department of Neurology, Vanderbilt University, Nashville, TN. 37240–7915. USA; 2 The Graduate Program of Neuroscience, Vanderbilt University, Nashville, 37240–7915. TN USA; 3 Department of Pediatrics, Peking University First Hospital, Beijing, 100034 China; 4 Present Address: University of Michigan, Life Sciences Institute, 210 Washtenaw Ave., Room 6115, Ann Arbor, MI, 48109-2216 USA

## Abstract

GABA_A_ receptors are brain inhibitory chloride ion channels. Here we show functional analyses and structural simulations for three *de novo* missense mutations in the GABA_A_ receptor β3 subunit gene (*GABRB3*) identified in patients with early-onset epileptic encephalopathy (EOEE) and profound developmental delay. We sought to obtain insights into the molecular mechanisms that might link defects in GABA_A_ receptor biophysics and biogenesis to patients with EOEE. The mutant residues are part of conserved structural domains such as the Cys-loop (L170R) and M2-M3 loop (A305V) that form the GABA binding/channel gating coupling junction and the channel pore (T288N), which are functionally coupled during receptor activation. The mutant coupling junction residues caused rearrangements and formation of new hydrogen bonds in the open state, while the mutant pore residue reshaped the pore cavity. Whereas mutant coupling junction residues uncoupled during activation and caused gain of function, the mutant pore residue favoured low conductance receptors and differential sensitivity to diazepam and loss of function. These data reveal novel molecular mechanisms by which EOEE-linked mutations affect GABA_A_ receptor function.

## Introduction

GABA_A_ receptors are heteropentameric GABA-gated chloride ion channels formed most commonly by the coassembly of 2α, 2β, and 1γ subunits, which mediate the majority of fast inhibitory neurotransmission in the brain. GABA_A_ receptor β3 subunits are widely expressed in the developing and adult brain^1^ in circuits involved in seizure generation such as cortex, hippocampus and thalamic reticular nucleus, where they mediate phasic and tonic inhibition^2^. Somewhat surprisingly, heterozygous *Gabrb3* ^+/−^ knock-out mice only exhibit mild absence-like seizures^3^.

Early onset epileptic encephalopathies (EOEE) are a group of heterogeneous epilepsy disorders that are almost invariable associated with poor prognosis and are treatment-refractory^4^. In general the syndromes are diagnosed in the first years of life, characterized by the onset of multiple seizure types, and associated with cognitive regression and intellectual disability. Whole-exome sequencing data indicated that sporadic *de novo* mutations were a major cause of these disorders^5^. In less than a decade, targeted gene testing has resulted in increasing reports of *de novo* GABA_A_ receptor gene (*GABR*) mutations associated with severe encephalopathies. A total of 19 likely pathogenic *de novo* missense mutations associated with EOEE have been identified in the gene encoding the GABA_A_ receptor β3 subunit (*GABRB3*)^6–10^, and *de novo* mutant residues in the β3 subunit were associated with West (Infantile Spasms) and Lennox-Gastaut syndromes, and with a broad phenotypic range of EOEE. Here we report the functional effects of three *de novo* β3 subunit mutant residues associated with EOEE, L170R^8^ (Cys-loop) and A305V^8^ (M2-M3 loop) that are located in the binding/gating coupling junction and a novel mutant pore residue, T288N (this study). The mutant residues are located in part of a structural core of the GABA_A_ receptor that is functionally coupled during GABA_A_ receptor activation.

Since *GABRB3* has been reported to have a variety of mutations associated with diverse forms of epileptic encephalopathy syndromes, we speculated that the occurrence of the pathogenic mutant residues within conserved structural domains of GABA_A_ receptors correlates with the dysfunction and the severity of the epileptic phenotype. *In vitro* studies reported that the GABA_A_ receptor function was differentially disrupted by the *de novo* β3 subunit mutant residues, D120N^11^, E180G^11^, Y184H^9^, L256Q^9^ and Y302C^9,11^, located at the GABA-binding interface and transmembrane domain that underlies channel activation, and these in turn were associated with the most severe forms of the EOEE epilepsy spectrum. We found that the impacts of the β3 subunit mutant residues L170R, T288N, and A305V on GABA_A_ receptor function and biogenesis were quite different, and it was entirely dependent on the location of the mutant residue in the highly conserved regions of the GABA_A_ receptor. These mutant residues occurred at the junction between the N-terminal region and the transmembrane domain of the receptor, which is the coupling junction, and in the pore domain of the receptor. These are conserved structural domains of all pentameric ligand-gated ion channels that couple conformational changes between the two structural domains upon agonist binding. Here, we found that the mutant residues in this region uncouple channel activation mainly through perturbations in the coupling junction and the pore, and that these are the molecular mechanisms that underlie the epilepsy syndrome phenotype.

## Results

### Three de novo mutations in GABRB3 were found in cases with EOEE

**Figure 1:**
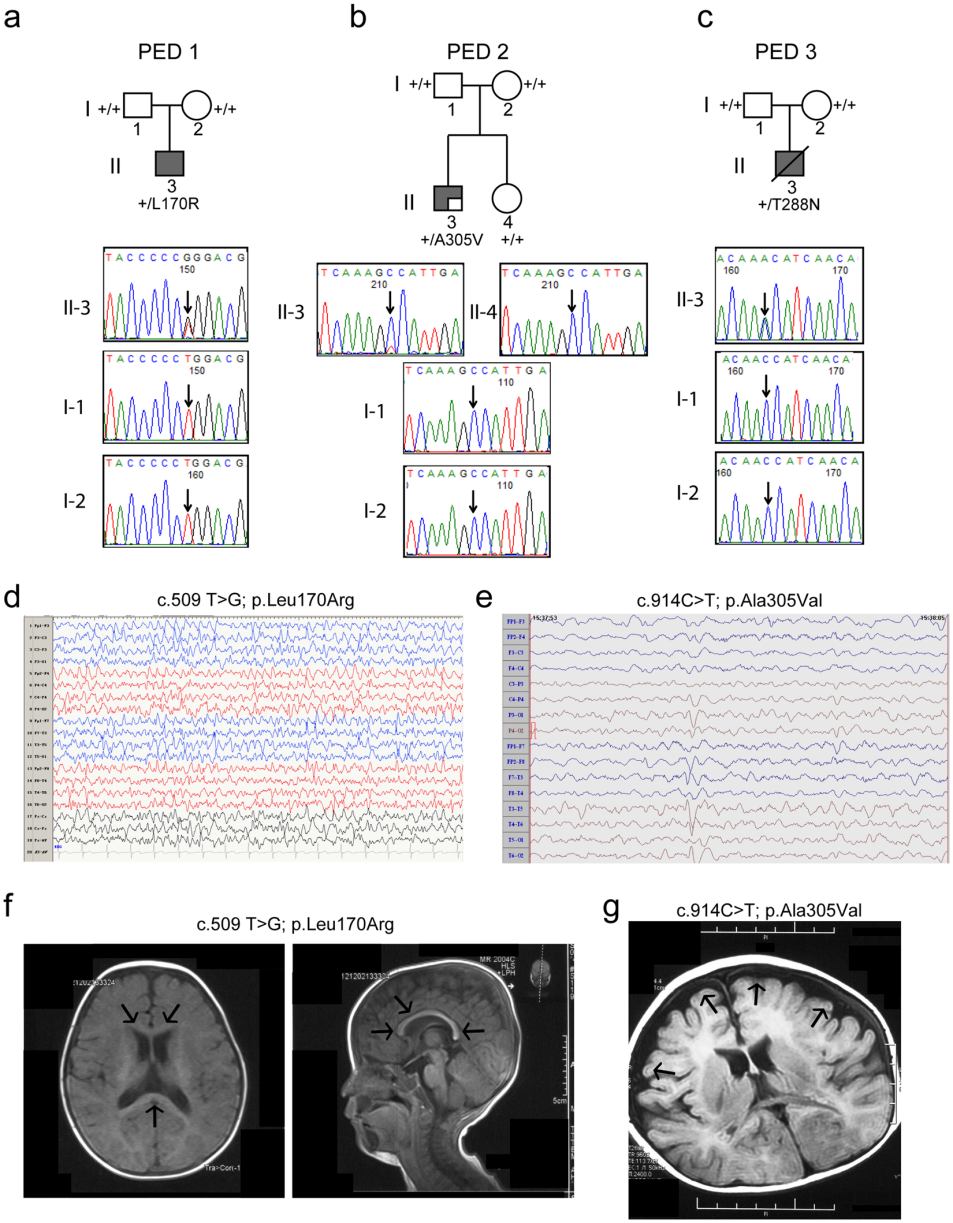
Trio-based sequencing analyses of families with de novo mutations in GABRB3. Pedigrees of the (**a**) L170R (PED 1), (**b**) A305V (PED 2), and (**c**) T288N (PED 3) *GABRB3* missense mutations in three affected probands. Chromatograms from the Sanger sequencing of the proband (II-3) displaying the *de novo* mutation and their unaffected father (I-1), mother (I-2) and dizygotic twin sibling (II-4) resulted from a dichorionic and diamniotic pregnancy. The site of the mutation was indicated by the arrows and compared with the NCBI reference gene (NM_021912.4). EEG samples of proband with (**d**) *GABRB3*(L170R) and (**e**) *GABRB3*(A305V) mutations were presented. During an interictal (sleep) period, proband PED 1-II-3 presented frequent multifocal spikes, and paroxysmal sharp wave discharges in the anterior region, and proband PED 2-II-3 presented generalized multifocal spikes of variable amplitude, and spike-slow wave complexes sporadically in the left occipital and posterior temporal regions. (**f**,**g**) Two patients reported nonspecific structural abnormalities in their MRIs. (**f**) Post contrast axial (left panel) and sagittal T1 (right panel) images of the brain of proband PED 1-II-3 that showed evidence of a thin corpus callosum (arrows); (**g**) while an axial image of proband PED 2-II-3 showed cortical atrophy (arrows) of the frontal lobes.

Previously we identified two unrelated patients with EOEE^8^, one who was heterozygous for the β3 subunit mutation L170R (c.509 T > G; p.Leu170Arg) and one who was mosaic for the β3 subunit mutation A305V (c.914 C > T; p.Ala305Val) with a frequency of the wild type allele (G) to the mutant allele (T) of 76/24. A recent screen found another unrelated patient with EOEE who was heterozygous for β3 subunit mutation T288N (c.863 C > A; p.Thr288Asn) (Fig. 1a–c). Functional studies have not been reported for any of these mutations. The clinical features of the three patients with the *GABRB3* mutations were summarized in Table S1, and representative EEG and brain MRI images in two patients were shown in Fig. 1d–g. The age of onset of epilepsy was within the first year of life in all three patients (3 to 6 months of age). Seizure semiology at onset was described as focal and secondary generalized tonic-clonic seizures in two patients (PED 1 and 2), and partial seizures and eyelid myoclonus in one patient (PED 3). EEG at 1 year showed generalized fast-waves in background activity, multifocal sharp and spike discharges during sleep in two patients (Fig. 1d,e). Developmentally, all three patients had severe intellectual disability, were non-verbal, and had severe motor disabilities at 1 year. All patients progressed to severe motor and cognitive impairment. Physical and neurological examinations were remarkable for the presence of hypotonia and poor coordination. One patient (PED 3) died at 18 months as a result of the severe psychomotor deficits and primary central nervous system failure. Brain MRIs showed mild nonspecific findings in two patients (thin corpus callosum, and cortical atrophy) (Fig. 1f,g) (Table S1).

### All three mutant β3 subunits reduced GABA-gated currents

**Figure 2:**
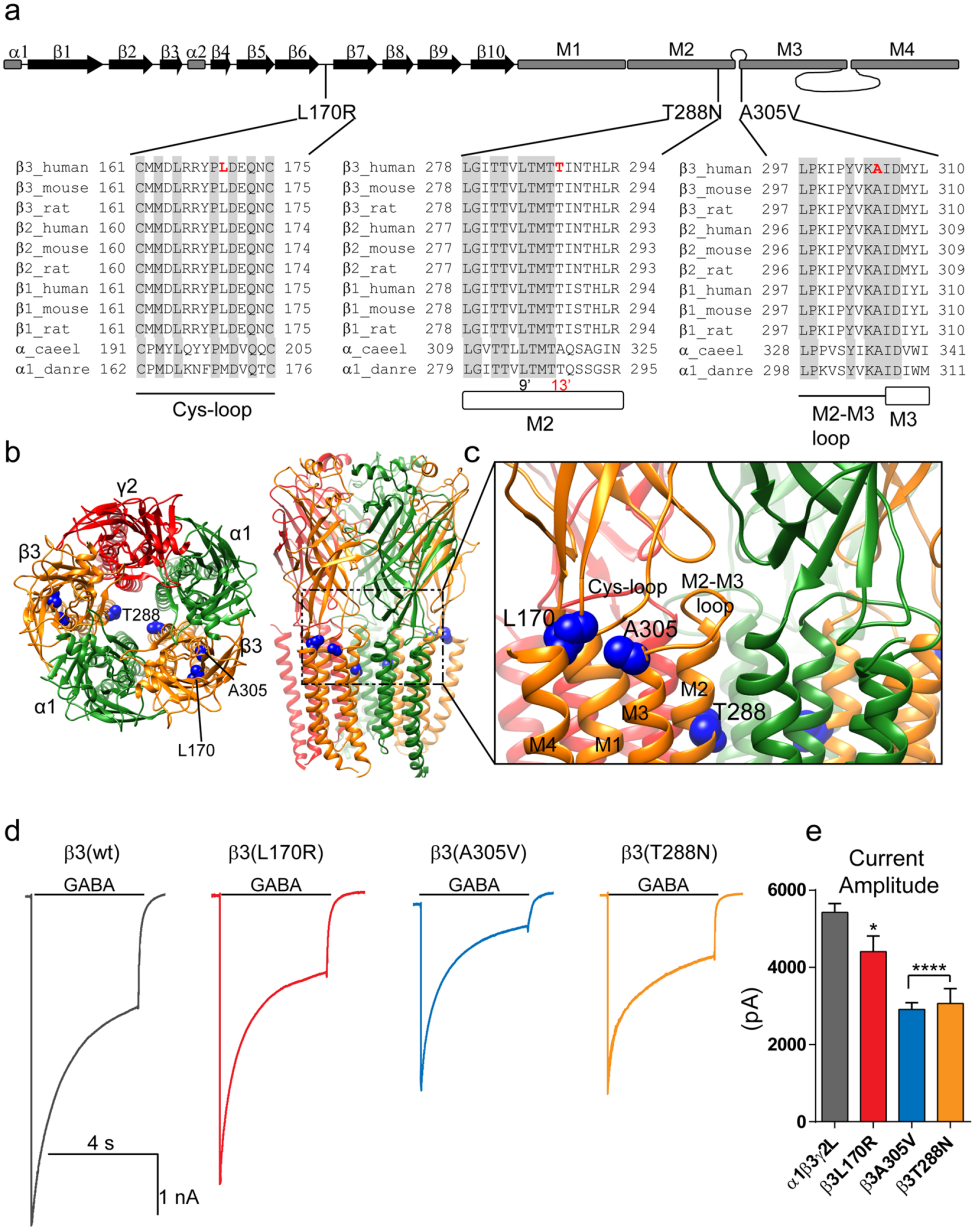
β3 mutations located at the junction between the extracellular region and the transmembrane domain decreased GABA_A_ receptor current amplitude. (**a**) Cartoon representation of the linearized secondary structure of the GABA_A_ receptor β3 subunit displaying the locations of the mutations. β-strands were represented as black arrows and α-helices as gray rectangles. Human, mouse and rat β(1–3) subunits from the GABA_A_ receptor family, and α subunits from the glutamate-gated chloride channel (α_caeel) and the glycine receptor (α1_danre) were aligned. Sites of *de novo* mutations in the β3 subunit were shown in red. Across all sequences, A305 is an identical residue (light gray), and L170 and T288 residues were conserved. L170 is located in the Cys-loop domain, and T288 and A305 in the transmembrane domains M2 and M3, respectively. T288 is at the 13′ position in the M2 domain (shown in red). The residues highlighted in grey were conserved across all of the subunits and pore-lining residues were numbered according to the protein sequence and position in the M2 domain. (**b**) 3D structural model of the α1β3γ2 GABA_A_ receptor in the open conformational state was shown as viewed from the N-terminal extracellular (left) and transmembrane (right) sides and displaying the β subunits in orange, α subunits in green and the γ subunit in red. The β3 subunit mutations were mapped onto the structure and represented in blue. The dashed box enclosed the junction between the extracellular region and the transmembrane domain of the receptor. (**c**) Enlarged view of the coupling junction and the pore domain highlighting the close location of the β3 mutations with structural domains at the interface of the N-terminal (Cys-loop) and transmembrane domains (M2-M3 loop, M1, M2, M3, M4) were indicated. (**d**) Representative GABA-gated current traces were obtained following rapid application of 1 mM GABA for 4 s to lifted HEK293T cells voltage clamped at −20mV expressing wild-type (wt) β3 and mutant β3(L170R), β3(A305V), and β3(T288N) subunit-containing α1β3γ2L GABA_A_ receptors. (**e**) Bar graphs summarized GABA-gated currents from cells expressing wild-type and mutant GABA_A_ receptors. Values were expressed as mean ± S.E.M. One-way ANOVA with Dunnett’s post-test was used to determine significance compared to the wild-type condition. ****p < 0.0001, and *p < 0.05, respectively.

By comparing the sequence alignments among GABA_A_ receptor β subunits from multiple species, the *Caenorhabditis elegans* glutamate-gated chloride channel (GluCl, α_caeel) and the *Danio rerio* glycine receptor α1 subunit (GlyRα, α1_danre) (Fig. 2a), we found that the mutant residues were mapped on conservative residues across the subunits in conserved structural domains of the β3 subunit. The substitutions to arginine, valine and asparagine were predicted to be damaging by SIFT with scores of 0.00, 0.00 and 0.01^12^ and PolyPhen2 with HumVar scores of 0.995, 0.998 and 0.983^13^. The mutant residues were located in major structural domains such as the Cys-loop (L170R), the M2-M3 loop (A305V), and the transmembrane domain 2 (M2, T288N) (Fig. 2b,c). The Cys-loop and the M2-M3 loop converge at the interface between the N-terminal domain and the pore domain, facing the pore domain and forming the coupling junction of the receptor. The five subunit M2 domains contribute the α helices that form the conducting pore of the channel. These regions are involved in receptor assembly and the GABA binding/channel gating coupling mechanism of GABA_A_ receptors^14–21^.

To determine how receptor function was affected, whole-cell currents were recorded from lifted HEK293T cells cotransfected with α1 and γ2L subunits and wild type (wt) β3 or coupling junction mutant β3(L170R) and β3(A305V) or the pore mutant β3(T288N) subunits by applying a saturating GABA concentration (1 mM) for 4 s using a rapid exchange system (Fig. 2d). Peak GABA-gated current amplitudes were significantly reduced in cells expressing mutant β3(L170R), β3(A305V) and β3(T288N) subunits to ~80%, 50%, and 60% of those expressing wt β3 subunits (Figure 2e; Table 1).

**Table 1:** Effects of GABA_A_ receptor β3 mutations L170R, A305V, and T288N on α1β3γ2L receptor channel function.

	***α1β3γ2L***	***α1β3L170Rγ2***	***α1β3A305Vγ2***	***α1β3T288Nγ2***
*Current amplitude, pA*	5426 ± 229 pA, n = 15	4408 ± 406 pA, n = 11, *p* < 0.05	2907 ± 178 pA, n = 10, *p* < 0.0001	3065 ± 385 pA, n = 9, *p* < 0.0001
*Desensitization extent, %*	66 ± 1%, n = 15	73 ± 2%, n = 11, *p* < 0.01	83 ± 2%, n = 10, *p* < 0.0001	69 ± 2%, n = 9
*Desensitization τ, ms*	1236 ± 85 ms, n = 15	808 ± 24 ms, n = 11, *p* < 0.0001	857 ± 36 ms, n = 10, *p* < 0.001	1319 ± 80 ms, n = 9
*Zinc inhibition, %*	13 ± 2%, n = 14	20 ± 1%, n = 11	42 ± 4%, n = 10, *p* < 0.0001	18 ± 2%, n = 9
*Activation τ, ms*	0.94 ± 0.03 ms, n = 21	1.25 ± 0.14 ms, n = 11, *p* < 0.05	1.85 ± 0.13 ms, n = 13, *p* < 0.0001	0.61 ± 0.03 ms, n = 10, *p* < 0.05
*Deactivation τ, ms*	110 ± 3.96 ms, n = 13	145 ± 2.23 ms, n = 11, *p* < 0.01	176 ± 12.5 ms, n = 13, *p* < 0.0001	106 ± 3.33 ms, n = 10

Values reported were mean ± S.E.M. One-way ANOVA with Dunnett’s multiple comparisons test was used to determine significance. **** *p* < 0.0001, *** *p* < 0.001, ** *p* < 0.01 and * *p* < 0.05, respectively, relative to α1β3γ2L.

### Coupling junction mutant β3(L170R) and β3(A305V) subunits reduced surface levels of GABA_A_ receptors

**Figure 3:**
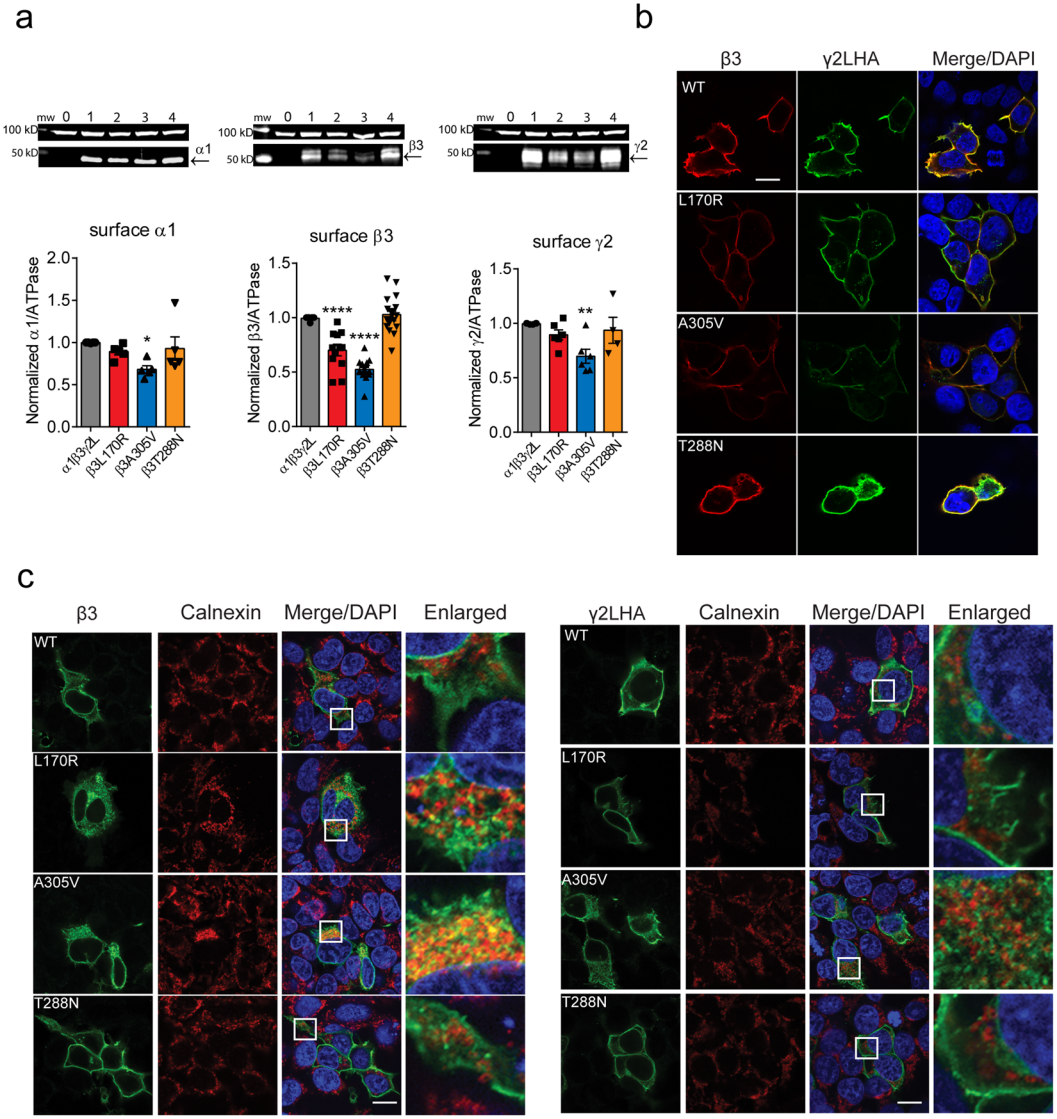
β3 mutant receptors reduced surface levels of GABA_A_ receptors subunits. (**a**) Wt β3 (lane 1) and mutant β3(L170R), β3(A305V), and β3(T288N) (lanes 2, 3, and 4, respectively) subunits were coexpressed with α1 and γ2L subunits in HEK293T cells, and surface protein samples were collected through surface biotinylation. Band intensity of surface expressed α1, β3 and γ2 subunits were normalized to the ATPase signal, and the summarized data were shown in the bar graphs. Values were expressed as mean ± S.E.M. One-way ANOVA with Dunnett’s post-test was used to determine significance compared to the wt condition. ****p < 0.0001, **p = 0.011, and *p = 0.048, respectively. Full-length gels were shown in supplementary Figure 3. (**b**) Wild-type or mutant β3 subunits were coexpressed with α1 and γ2L^HA^ subunits in HEK293T cells. Surface staining patterns were revealed by confocal microscopy. Non-permeabilized cells were stained with antibodies against the β3 subunit (red) and the HA tag (green). Scale bars, 10 μm. (**c**) The transfected cells were permeabilized, and β3 and γ2L^HA^ subunits were labelled with anti-β3 and anti-HA antibodies, respectively (green). The ER was visualized with anti-calnexin antibody (red). White boxes on the merged images depict the enlarged area shown in the images to the right. Scale bars, 20 μm. Also shown were DAPI nuclear counterstaining (blue) and the merge of the stainings.

To gain insights into whether the mutant β3 subunits reduced GABA-gated currents due to loss of GABA_A_ receptors expressed on the cell surface membrane, we coexpressed α1, γ2L and either wt β3 or mutant β3(L170R), β3(A305V) or β3(T288N) subunits in HEK293T cells and assessed surface expression levels of β, γ and α subunits (Fig. 3a).

The coupling junction mutant β3(L170R) and β3(A305V) subunits altered the surface expression patterns of α1, β3 and γ2L subunits differently. Coexpression of β3(L170R) with α1 and γ2L subunits resulted in a significant reduction of surface β3 subunit levels to about 70% of control levels (0.70 ± 0.05, n = 12, *p* < 0.0001) with lack of effect on surface α1 (0.89 ± 0.04, n = 5) or γ2L (0.90 ± 0.05, n = 6) subunit levels. In contrast, coexpression of β3(A305V) with α1 and γ2L subunits significantly decreased surface α1 (0.68 ± 0.04, n = 5, *p* = 0.048), β3 (0.52 ± 0.03, n = 12, *p* < 0.0001) and γ2L (0.70 ± 0.06, n = 6, *p* = 0.011) subunit surface levels to 50–70% of control levels. Not surprisingly the pore mutant β3(T288N) subunit did not cause any change in the overall pattern of surface expression of GABA_A_ receptor subunits (α1: 0.93 ± 0.14, n = 5; β3:1.03 ± 0.04, n = 16; γ2L: 0.94 ± 0.12, n = 4).

In contrast, none of the mutant β3 subunits reduced total levels of α1 (L170R, 1.05 ± 0.05, n = 4, A305V, 1.01 ± 0.05, n = 4, T288N, 0.97 ± 0.03, n = 4, *p* = 0.5984), β3 (L170R, 0.99 ± 0.08, n = 4, A305V, 0.94 ± 0.04, n = 4, T288N, 1.12 ± 0.05, n = 4, *p* = 0.1474), or γ2L (L170R, 0.94 ± 0.08, n = 4, A305V, 0.91 ± 0.03, n = 4, T288N, 1.01 ± 0.05, n = 4, *p* = 0.4827) subunits.

### Coupling junction mutant β3(L170R) and β3(A305V) subunits reduced receptor trafficking to the cell surface

To determine where in the cells the coupling junction mutant β3(L170R) and β3(A305V) subunits and the pore mutant β3(T288N) subunit were trafficked, wt or mutant β3 subunits were coexpressed in HEK293T cells with α1 and γ2L^HA^ subunits, and the cellular locations of mutant β3 subunits were assessed using confocal microscopy (Fig. 3b). Thus, without cell permeabilization, the cells were colabeled with anti-β3 subunit (red) and anti-HA (green, γ2L subunits) antibodies to detect subunits expressed on the cell surface. All three mutant β3 subunits were present on the cell surface and were colocalized with γ2L^HA^ subunits, consistent with coassembly of γ2L and β3 subunits into receptors that were trafficked to the cell surface. However, only the coupling junction mutant β3(L170R) and β3(A305V) subunits had reduced surface β3 (both mutant residues) and/or γ2L (only A305V) subunits. These results corroborated the results found by surface biotinylation and demonstrated that the coupling junction mutant β3(L170R) and β3(A305V) subunits produced defects in trafficking of mutant β3 subunit-containing receptors, whereas the pore mutant β3(T288N) subunit did not affect GABA_A_ receptor trafficking.

### Coupling junction mutant β3(L170R) and β3(A305V), but not the pore mutant β3(T2888N), subunits were partially retained in the ER

We hypothesized that coupling junction mutant β3(L170R) and β3(A305V), but not the pore mutant β3(T2888N), subunits were subject to ER retention, leading to reduced surface receptor expression. Thus, permeabilized HEK293T cells coexpressing wt or mutant β3 subunits with α1 and γ2L^HA^ subunits were colabeled with anti-β3 subunit or anti-HA and anti-calnexin antibodies (Fig. 3c). The latter was used as an ER marker. Calnexin exhibits a typical perinuclear and reticular distribution inside the ER, consistent with its role as a constituent of the ER protein quality control mechanism that recognizes and retains mutant proteins and components of misassembled proteins^22^. Wild-type β3 and γ2L^HA^ subunits spread outside the ER, which suggested the presence of newly synthesized subunits that were in transit to the cell surface. Similarly the pore mutant β3(T288N) subunit had an intracellular distribution resembling that of wt β3 and γ2L^HA^ subunits. In contrast, the coupling junction mutant β3(L170R) and β3(A305V) subunits were mainly colocalized with calnexin, and thus were retained in the ER.

### Pharmacological properties of GABA_A_ receptors containing each mutant β3 subunit

So far the results indicated that the coupling junction mutant β3(L170R) and β3(A305V) subunits reduced the trafficking of GABA_A_ receptor subunits to the cell surface, thereby reducing GABA-gated currents. However, this correlation did not occur for the pore mutant β3(T288N) subunit, which had decreased GABA-gated currents with no reduction of surface GABA_A_ receptors. As all three β3 subunits had mutant residues located in strategic regions of the receptor that determine the transduction of the binding of ligands from the N-terminal domain to the pore^16–21^, we hypothesized that the sensitivities to GABA, diazepam (DZP) and Zn^2+^ were affected differently depending on the location of the mutant residue in the GABA_A_ receptor. The binding sites for these compounds have been widely studied and are distributed in the extracellular region and the transmembrane domains of the receptor^23–28^.

**Figure 4:**
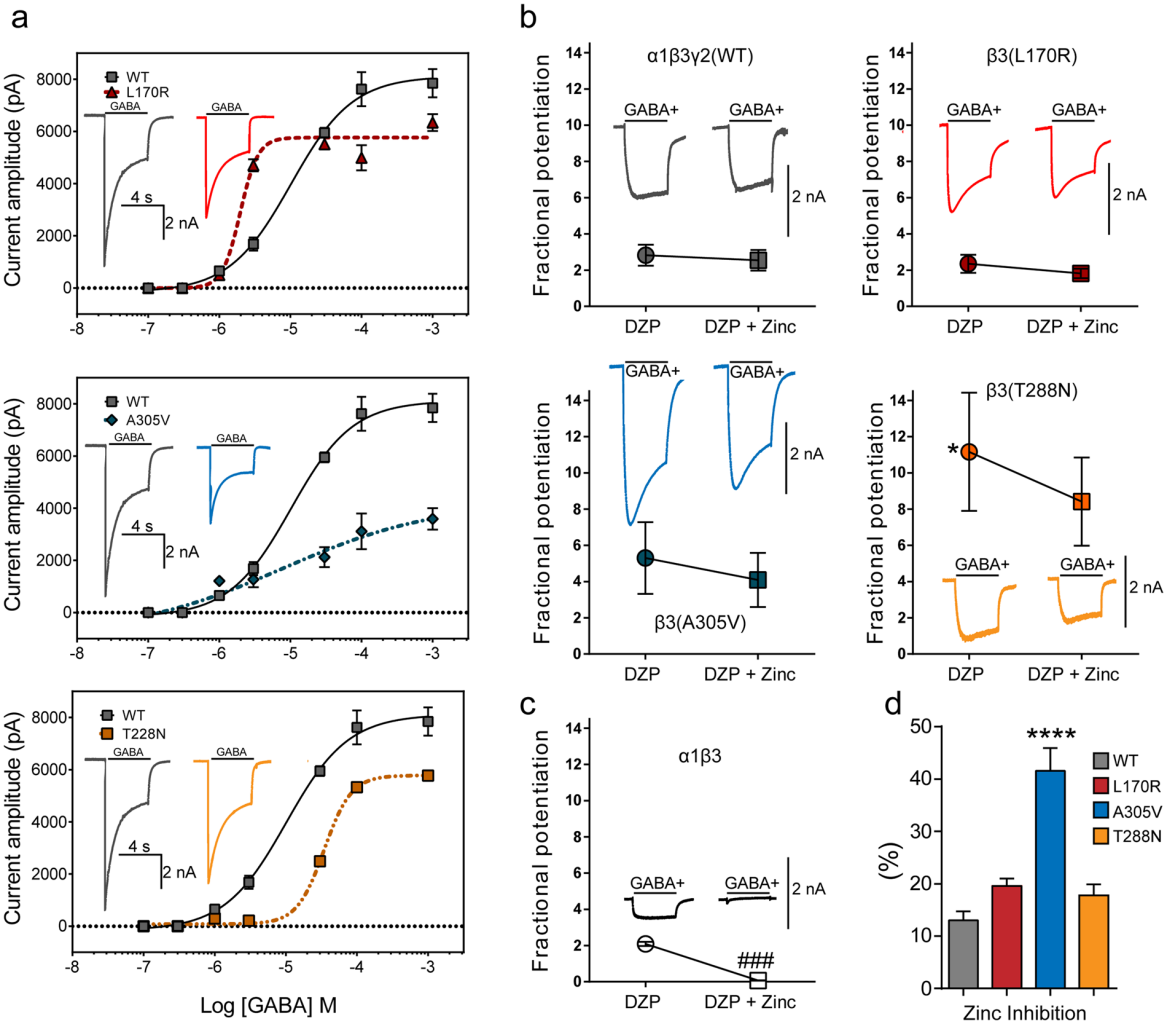
The β3 subunit mutations altered the sensitivity of GABA_A_ receptors to GABA, DZP and Zn^+2^. (**a**) Concentration-response curves for wt β3 (solid lines) and mutant β3(L170R), β3(A305V), and β3(T288N) (dashed lines) subunit-containing α1β3γ2L GABA_A_ receptors were obtained. Inside the panels, representative peak currents evoked by a 4 s application of GABA (100 μM) were shown. The color of the traces indicated the experimental condition as represented in the GABA_A_ receptor concentration curves. Amplitude/time scale bars for currents from GABA_A_ receptors containing wt and mutant β3 subunits were 2 nA/4 s. Values were expressed as mean ± S.E.M (n = 5–6 cells for each experimental condition). The data represented the summary of 24 cells with comparable capacitances (8–12 pF) recorded from three independent transfections. (**b**) Effect of 1 µM DZP (filled circles) and 1 µM DZP + 10 µM Zn^2+^ (filled squares) on GABA-gated currents produced by 4 s applications of 1 µM GABA to (wt) β3 and mutant β3(L170R), β3(A305V), and β3(T288N) subunit-containing α1β3γ2L GABA_A_ receptors. Representative peak currents for each experimental condition were shown inside the panels. (**c**) Effect of 1 µM DZP (open circle) and 1 µM DZP + 10 µM Zn^2+^ (open square) on GABA-gated currents produced by 4 s applications of 1 µM GABA to (wt) α1β3 receptors. Above the symbols were shown representative GABA-gated currents. (**d**) Bar graphs summarize 1 mM GABA-gated currents in the presence of 10 µM Zn^2+^ from cells expressing wt and mutant GABA_A_ receptors. Values were expressed as mean ± S.E.M. One-way ANOVA with Dunnett’s post-test was used to determine significance compared to the wild-type condition. ****p < 0.0001, and *p < 0.05, respectively. See Table 2 for details.

To measure changes in GABA potency and efficacy of wt and mutant α1β3γ2L GABA_A_ receptors, we measured the effects of the mutant β3(L170R), β3(A305V), and β3(T288N) subunits on GABA concentration-response curves (Fig. 4a). Peak GABA-gated currents were obtained by applying increasing concentrations of GABA for 4 s to wt α1β3γ2L and mutant α1β3(L170R)γ2L, α1β3(A305V)γ2, and α1β3(T288N)γ2L GABA_A_ receptors. For wt receptors, GABA EC_50_ was 10.2 ± 1.23 µM, and the maximal current was 8117 ± 167 pA (n = 5–6). In the presence of the mutant β3 subunits, the receptor’s response to GABA varied considerably depending on the location of the mutant residue. Thus, the coupling junction mutant subunits either caused a ~5-fold increase (L170R) or a small increase (A305V) in GABA_A_ receptor potency, and both mutant subunits reduced the maximal response to GABA to 40–70% of control currents (Table 2). Conversely, the pore mutant β3(T288N) subunit caused a ~3.5-fold decrease in GABA_A_ receptor potency, with a reduction to 70% of control response of the maximal response to GABA (Table 2).

**Table 2:** Potentiation of GABA-gated currents by GABA, diazepam and diazepam in presence of Zn^2+^.

	***Current activation by GABA*** ^1^ ***(I***_***MAX***_^2^)	***Potentiation by diazepam***	***Potentiation by diazepam in presence of Zn*** ^***2+***^	***p*** ^***3***^
*α1β3γ2*	10.2 ± 1.23 (8117 ± 167)	2.82 ± 0.58	2.53 ± 0.57	0.7333 (n = 5)
*α1β3L170Rγ2*	1.97 ± 1.12 (5764 ± 152)	2.35 ± 0.50	1.82 ± 0.26	0.3912 (n = 4)
*α1β3A305Vγ2*	6.87 ± 1.58 (3371 ± 213)	5.30 ± 1.98	4.09 ± 1.49	0.6381 (n = 5)
*α1β3T288Nγ2*	34.6 ± 1.04 (5781 ± 87.2)	11.2 ± 3.26*	8.41 ± 2.43	0.5204 (n = 5)
*α1β3*	ND	2.09 ± 0.12	0.07 ± 0.01	0.0004 (n = 4)

^1^GABA EC_50_ was expressed in µM, and ^2^I_MAX_ (maximal current) was expressed in pA (n = 5–6). Values represent mean ± S.E.M. ^3^Unpaired two-tailed Student’s t test relative to diazepam alone. One-way ANOVA with Dunnett’s multiple comparisons test was used for significance between wt α1β3γ2 and mutant GABA_A_ receptors or α1β3 relative to diazepam alone. *Indicate *p* = 0.0152.

Differences in GABA potency and efficacy can be explained by differences in GABA_A_ receptor subunit composition such as a switch from ternary αβγ receptors to binary αβ receptors. To determine whether the mutant β3 subunits altered GABA_A_ receptor subunit composition, we compared 1 µM GABA-gated current amplitudes in the presence of 1 µM DPZ or 1 µM DPZ + 10 µM Zn^2+^ (Fig. 4b). Thus, it is expected that ternary GABA_A_ receptors composed of αβγ subunits are potentiated by benzodiazepines and lack high affinity inhibition by Zn^2+^ while binary GABA_A_ receptors composed of αβ subunits are not potentiated by benzodiazepines but are inhibited by low concentrations of Zn^2+^. None of the mutant β3 subunits impaired DZP potentiation of GABA-gated currents (Fig. 4b, left filled circles). Remarkably, while the DZP potentiation of receptors containing the coupling junction mutant β3(L170R) and β3(A305V) subunits was similar to the potentiation of wt receptors (Table 2), the potentiation of receptors containing the pore mutant β3(T288N) subunit was increased by 4-fold (*p* = 0.015).

To further confirm that the DZP potentiation observed was due to interactions with ternary αβγ GABA_A_ receptors and not binary αβ receptors, DZP was coapplied with Zn^2+^ (Fig. 4b, right filled squares). If binary αβ receptors were present, Zn^2+^ would decrease the DZP potentiated current. Despite the fact that there was a trend for reducing the potentiating effect of DZP in the presence of Zn^2+^, the difference was not significant and appeared not to affect the potentiation by DZP (Table 2). In addition, when GABA-gated currents from αβ receptors were recorded in presence of DZP (Fig. 4c, left open circle), a similar response to that of αβγ receptors was found (Table 2). Nevertheless, in the presence of Zn^2+^, the αβ receptors DZP response was completely blocked (Fig. 4c, right open square). Moreover, the sensitivity to Zn^2+^ was affected differently depending on the specific mutant β3 subunit (Fig. 4d). Whereas receptors containing the mutant β3(L170R) and β3(T288N) subunits displayed fractional Zn^2+^ inhibitions that resembled wt GABA_A_ receptors, receptors containing the β3(A305V) subunit seemed to have a slight increase (~20%) in Zn^2+^sensitivity.

### Altered GABA_A_ receptor gating produced by mutant β3 subunits correlated with structural perturbations that depended on the conformational state of the receptor

**Figure 5:**
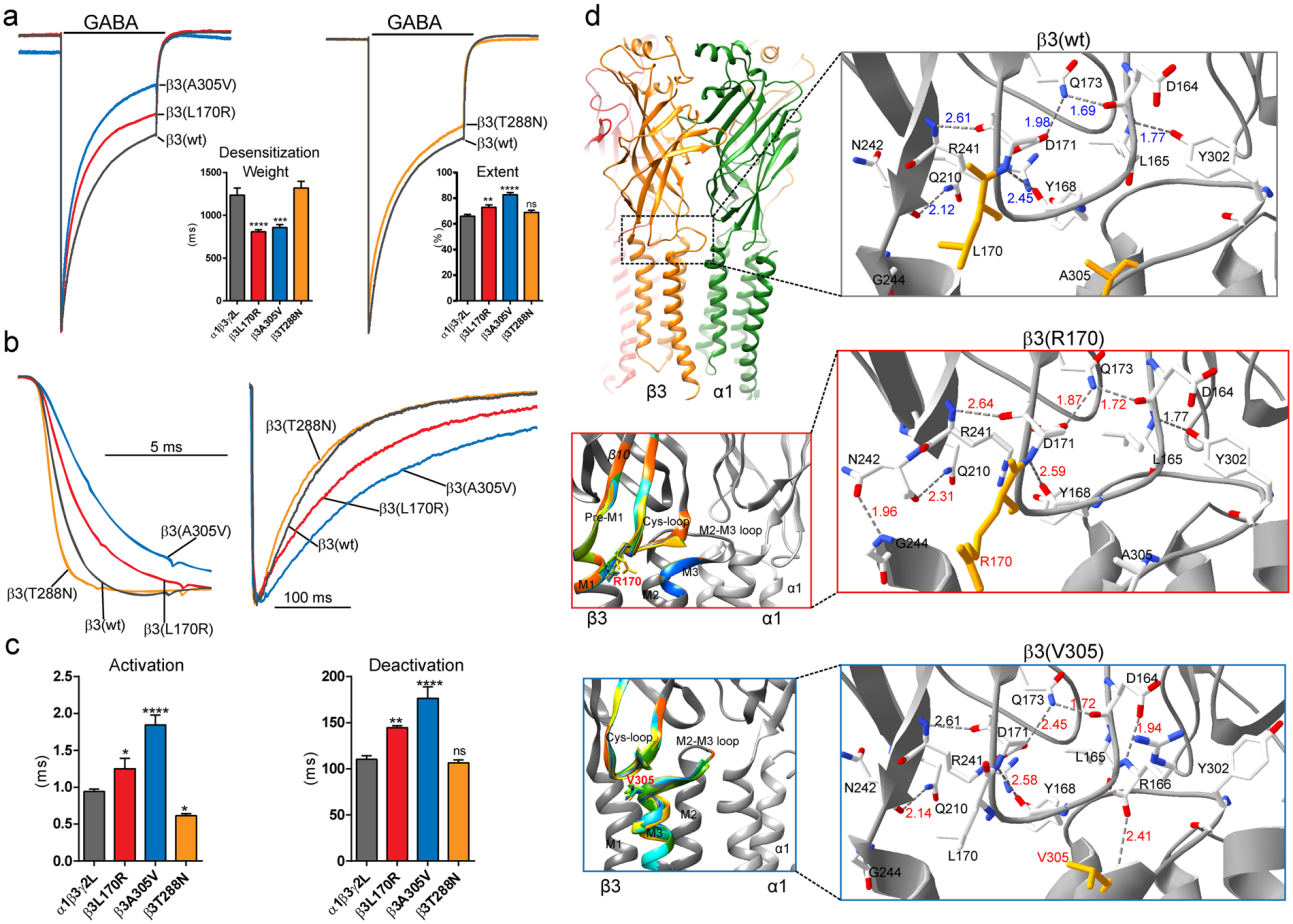
GABA_A_ receptors containing mutant β3 subunits had altered current kinetics that correlated with perturbations transmitted through canonical loops. (**a**) Representative current traces were superposed and normalized to control currents to show the desensitization time course and extent of desensitization of GABA-gated currents produced by 4 s applications of 1 mM GABA to wt β3 and mutant β3(L170R), β3(A305V), and β3(T288N) subunit-containing α1β3γ2L GABA_A_ receptors. Bar graphs to the right of traces show the weighted desensitization time constant (τ) during application of GABA, and the average extent of desensitization measured at the end of the application of GABA. (**b**) Representative normalized current traces showing activation and deactivation of currents produced by 10 ms GABA (1 mM) applications to wt and mutant receptors. (**c**) Bar graphs show average current activation and deactivation time constants from cells coexpressing α1 and γ2L subunits with wt or mutant β3 subunits. Values were expressed as mean ± S.E.M. One-way ANOVA with Dunnett’s post-test was used to determine significance. ****p < 0.0001, ***p < 0.001, **p < 0.01, *p < 0.05, and ^**ns**^ *p* > 0.05, respectively. See Table 1 for details. (**d**) On the upper left, 3D models of two wt neighbouring β (orange) and α (green) subunits in the open conformation were shown with the coupling junction enclosed by the dashed box. On the bottom left, enlarged views of the coupling junction show structural perturbations in the secondary structure of the β3 subunit that differed among wt (in gray) and mutant β3(L170R) and β3(A305V) subunit models (RMS deviation ≥ 0.5 Å were represented in a different colour). The structural domains along the coupling junction (β10-strand, Cys-loop, Pre-M1) and transmembrane domains (M2-M3 loop, M1, M2, M3) were indicated. On the right, enlarged views of the networking of hydrogen bonds of the β3 (wt) and mutant β3(L170R) and β3(A305V) subunit models predicted at the coupling junction in the open conformation. In stick representation, the wt and mutated residues at positions 170 and 305 were coloured orange. Hydrogen bond distances were given in Å, and labelled in red when they differed from the wt model. N, Asparagine. Q, Glutamine. R, Arginine. D, Aspartate. L, Leucine. G, Glycine. A, Alanine. V, Valine. Y, Tyrosine.

To gain insights into which of the macroscopic properties of GABA_A_ receptor currents were altered, we measured desensitization, activation and deactivation rates of GABA-gated currents by applying a saturating GABA concentration (1 mM) for 4 s (desensitization) and 10 ms (activation and deactivation) (Table 1). GABA-gated currents recorded from cells coexpressing α1 and γ2L subunits with coupling junction mutant β3(L170R) and β3(A305V) subunits were more rapidly and strongly desensitized (Fig. 5a), ~1.5–2-times more slowly activated, and ~1.3–1.6-times more slowly deactivated (Fig. 5b,c) than wt receptor currents (Table 1). In contrast, coexpression of the pore mutant β3(T288N) subunit only accelerated current activation (Fig. 5b,c), with no effects on desensitization or deactivation currents (Fig. 5a–c). These results suggested that the coupling junction mutant residues L170R and A305V located in the β3 subunit extracellular domain govern different kinetic processes during channel gating than the pore mutant residue T288N located in the transmembrane domain.

**Figure 6:**
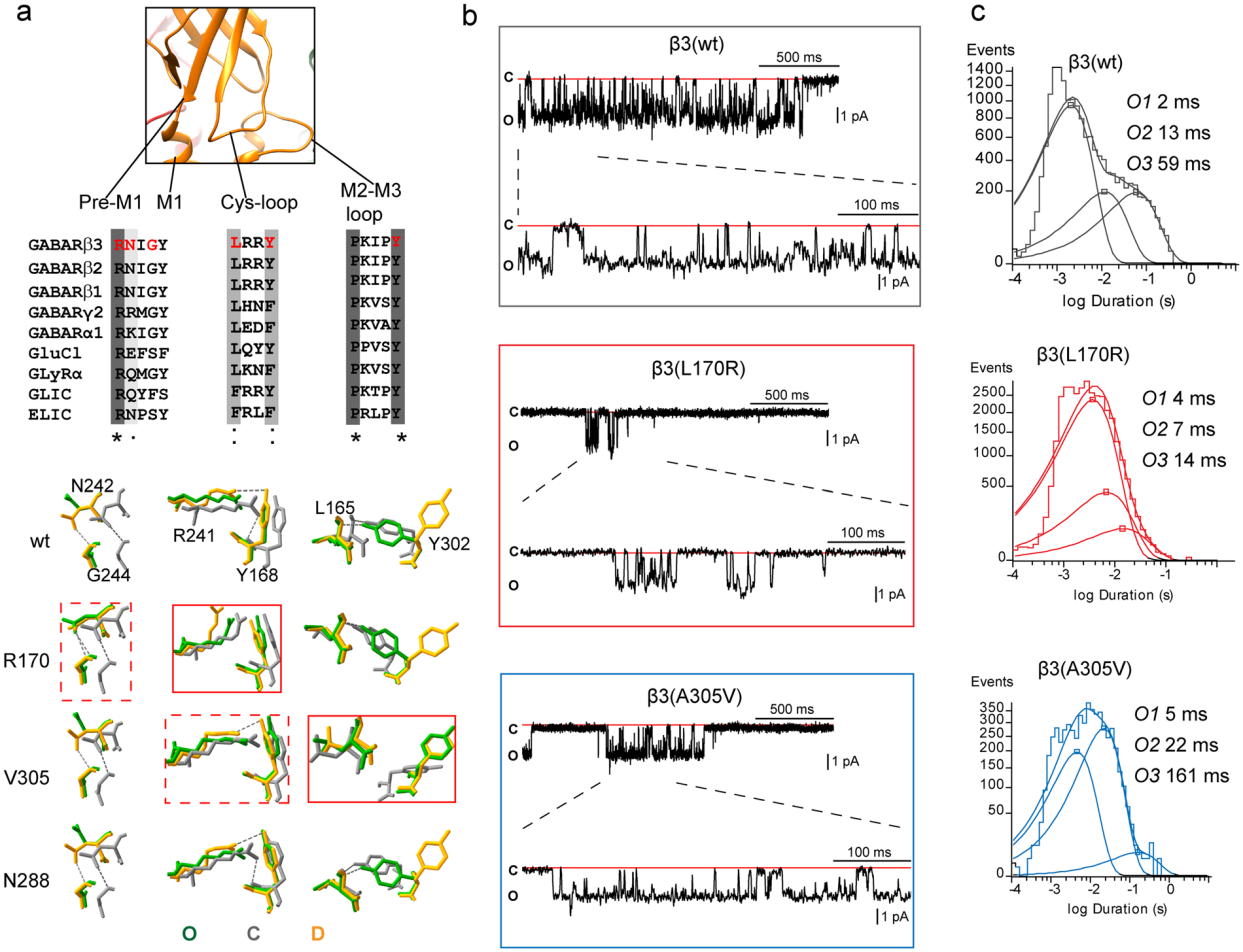
β3 subunit mutations differently altered GABA_A_ receptor single channel properties. (**a**) Enlarged view of the coupling junction of a β3 subunit model specifying the network of four bordering regions that predicted different H-bonds depending on the conformational state of the receptor. The location and conservation of the residues within the four regions were highlighted in red in the sequence alignments below (*identical.***:*** conservative.. semi-conservative). The network of interactions among resides located at the coupling junction was superimposed in stick representation and in different colors depending of the state of wt and mutant β3 subunit structures (C, closed state in gray, O, open state in green, and D, desensitized state in orange). Dashed boxes represented perturbations in solely one state, and solid boxes in all states (see details in text). (**b**) Single-channel GABA-gated current traces from patches with receptors containing wt β3 or mutant β3 L170R or A305V subunits with representative clusters of openings. Patches were voltage clamped at + 80 mV and continuously exposed to 1 mm GABA. Openings were downward and each representative trace was a continuous 2000 ms recording. (**c**) Open time histograms for wt and mutant receptors were fitted to three exponential functions. See Table 3 for details.

**Figure 7:**
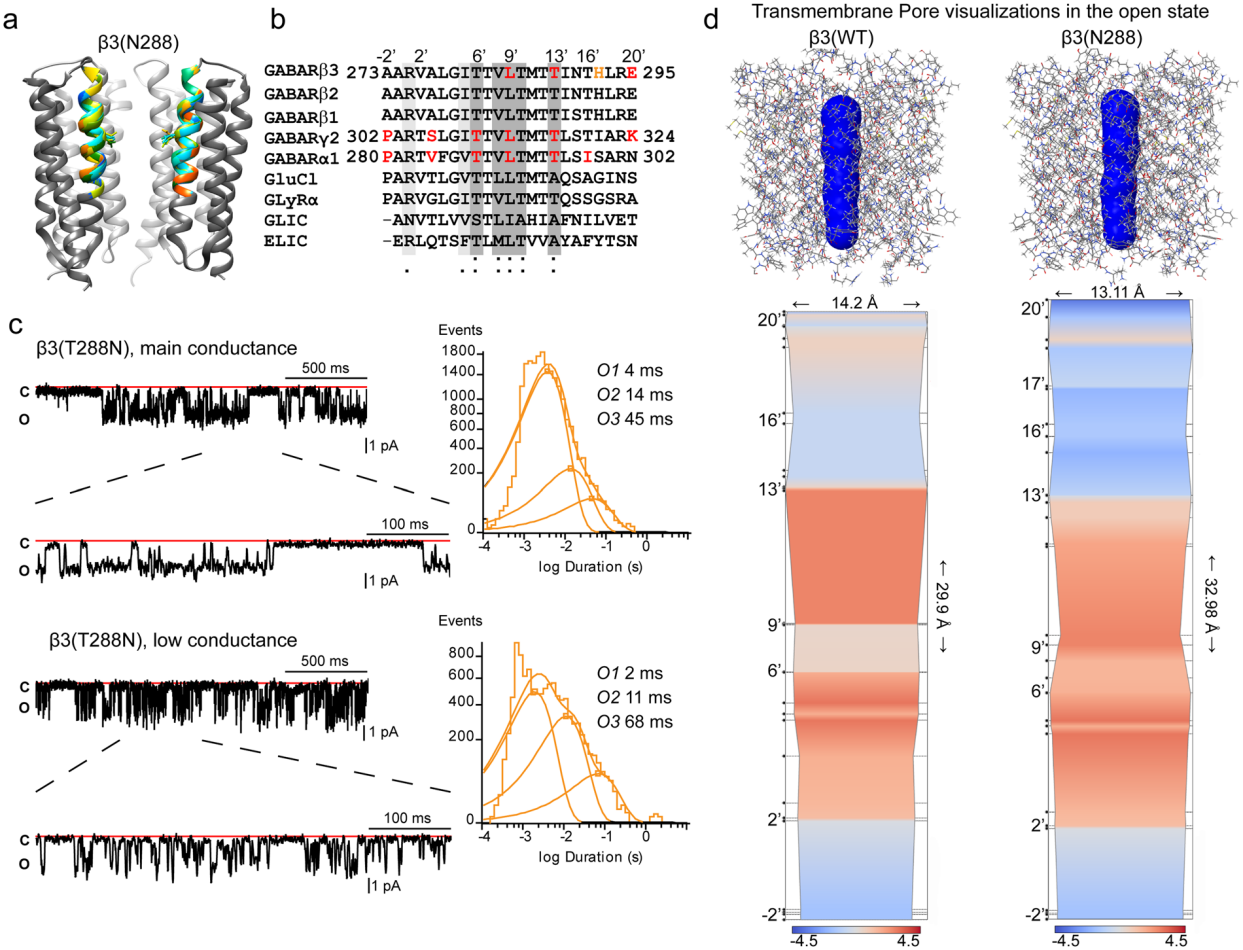
The β3 subunit pore mutation T288N destabilized channel openings by altering the GABA_A_ receptor conduction pathway. (**a**) Superimposed 10-best-scoring transmembrane domains of mutant T288N receptors in the open state were seen parallel to the membrane. One subunit was removed for clarity. Perturbed side chains of the pore-lining residue at 13′ position were shown within the ion channel pore, and the structural perturbations that differ among wt and mutant subunit (RMS ≥ 0.5 Å) were represented in different colors. The wt structure was in gray. (**b**) Alignments of the transmembrane M2 domain of GABA_A_ receptor, GluCl, glycine, GLIC, and ELIC receptors were made to highlight the evolutionary residue conservation as shown in Fig. 6. The residues were numbered according to protein sequence and position in M2. In red were highlighted pore-lining residues part of the inner face of the cavity predicted for both wt and T288N structures. In orange, β3H292 (17′ position) was predicted for the T288N structure. (**c**) Representative single-channel current traces from cell-attached patches of receptors containing mutant β3(T288N) subunits were recorded as shown in Fig. 6. Note that the cluster of openings fall into the two distinct types of conductance: main (upper panel), and low (lower panel) openings. C and O refer to closed and open states. Open time histograms were shown to the right of the panels. See Table 3 for details. (**d**) Skin representations (in blue) of the pore cavity of wt β3 and mutant β3(T288N) subunit containing receptors in the open state (in CPK representation) were made. Bottom panels show the hydrophilicity/hydrophobicity properties of the pore cavity of GABA_A_ receptors containing wt β3 and mutant β3(T288N) subunits in the form of 2D plots. The hydropathic profile produced by the pore-lining residues are represented as hydropathy scales between −4.5 (blue, hydrophilic side) and + 4.5 (red, hydrophobic side). Along the pore-axis, the positions of the pore-lining residues were identified.

It is well known that the coupling junction couples conformational changes between the GABA_A_ receptor GABA binding site in the N-terminal domain and the pore^16–21^. To shed light on whether perturbations in channel structure caused by the location of the β3 subunit mutant residues were correlated with the observed kinetic changes resulting in impairment of the transitions among the closed, open and desensitized states of the receptor, we generated wt and mutant pentameric αβγ GABA_A_ receptor models using the *Danio rerio* GlyRα^29^ in the three conformation states (open, closed and desensitized) (Fig. 5d, Fig. 6a, Fig. 7a, and supplemental Fig. 1) as templates (see Materials and Methods for details). We computed perturbations of the subunit’s secondary structure by computing the Root Mean Square (RMS) deviation between wt and mutant structural models. When the perturbations of the secondary structure (ribbon representation) had RMS deviation values ≥ 0.5 Å, they were shown in rainbow colors as a result of the superposition of the 10 best models (10 lowest-energy models out of 20). Our results demonstrated that the coupling junction mutant residues L170R and A305V caused similar disturbances that were propagated through the coupling junction, perturbing the Cys-loop, Pre-M1 region, M1, M3 and M2-M3 loop at the extracellular junction between the N-terminal domain and transmembrane domain in the open (Fig. 5d, left panels), closed, and desensitized states (supplemental Fig. 1a,b). Conversely, the pore mutant residue T288N altered mainly the transmembrane domain in the open state (Fig. 7a, and supplemental Fig. 1c).

Despite the similarity in alterations of the secondary structure caused by the coupling junction mutant residues L170R and A305V, distinct perturbations in the network of hydrogen bonds (H-bonds) between residues within the coupling junction were predicted, which suggested that specific local structural disturbances may be transduced through the residues that comprise the coupling junction to the entire subunit (Fig. 5d, right panels, and supplemental Fig. 1a,b). Hence we considered the network of adjacent interactions among residues that are located in the bordering regions at the coupling junction. We identified four nearby regions with their respective amino acids that predicted different H-bonds depending on the conformational state of the receptor. The four domains and their amino acids are represented in Fig. 6a, as follows: Pre-M1 (R241 and N242), M1 (G244), Cys-loop (L165 and Y168), and M2-M3 loop (Y302) (Fig. 6a. top panel).

In the open conformation, the wt model predicted an H-bond network between Y302 in the M2-M3 loop and L165 in the Cys-loop, while no interactions were predicted between the Pre-M1 and M1 domain or the Cys-loop (Fig. 5d, top right panel). In contrast, the L170R mutant residue formed an additional H-bond network between N242 in the Pre-M1 domain and G244 in M1, while the A305V mutant residue lacked the Y302-L165 network, and formed a novel H-bond between R166 in the Cys-loop and V305 in the M2-M3 loop (Fig. 5d, middle and bottom right panels). When analyzing the H-bond networks that were predicted between desensitized and closed states, the wt subunit lacked the Y302-L165 H-bond network in the desensitized stated, and showed the N242-G244 and R241-Y168 H-bond networks. In contrast, in the closed stated, the wt subunit displayed the three H-bond networks (supplemental Fig. 1a,b, top panels). For the coupling junction mutant residues L170R and A305V (supplemental Fig. 1a,b, middle and lower panels), L170R lacked the R241-Y168 H-bond network in both desensitized and closed states, while A305V caused small differences in the desensitized state, but lacked the R241-Y168 H-bond and Y302-L165 H-bond networks in the closed stated. Further, the pore mutant residue T288N did not change the network of interactions observed in wt subunits (supplemental Fig. 1c). A better view of the observed changes was obtained by the superposition of the three networks of residues at the coupling junction (Fig. 6a, bottom panel). In all conformational states, the L170R mutant residue primarily affected the R241-Y168 H-bond network, while the A305V mutant residue shifted the perturbations towards the Y302-L165 H-bond network of the channel (Fig. 6a, solid red boxes). Noteworthy, the N242-G244 H-bond network was solely affected by the L170R mutant residue in the open state, and the R241-Y168 H-bond network by the A305V mutant residue in the closed state (Fig. 6a, dashed red boxes).

In addition, we found that the perturbations of the network of H-bonds were correlated with structural rearrangements of sidechains among neighboring residues of the four domains at the coupling junction. We measured predicted disturbances caused by the mutant sidechain residues L170R, A305V and T288N at Pre-M1_M1 (R241-Y245), Cys-loop (L165-Y168), M2-M3 loop (P298-Y302), and M2 (T281-E295) domains in the open conformation (supplemental Fig. 4a), since those residues are known to be key for translating ligand binding to channel gating of the receptor. While L170R predicted the largest RMS deviation on Pre-M1_M1, A305V had the greatest effects on M2-M3 loop. Both L170R and A305V predicted larger RMS deviation on the Cys-loop, but A305V on a larger scale than L170R. As expected, the pore mutant residue T288N had exclusive effects on M2.

### Coupling junction β3 subunit mutant residues L170R and A305V enhanced GABA_A_ receptor gating

Our results demonstrated that the β3 subunit coupling junction mutant residues L170R and A305V appeared to increase the gating of the channel either by increasing GABA potency (L170R) and/or prolonging current deactivation (L170R and A305V), and these observations were correlated with specific structural changes at the coupling junction.

Since deactivation rate correlates with the average time channels are bound to GABA, representative microscopic measurements include open probability, mean open time, and burst duration. To determine which of these microscopic properties were affected by the β3 subunit mutant residue, we obtained single channel recordings from wt and mutant α1β3γ2L GABA_A_ receptor channels (Fig. 6b,c). As expected, wt receptors opened into brief bursts of openings and frequent prolonged (>500 ms) clusters of bursts to a main conductance level of ~25 pS in response to saturating concentrations of GABA^30,31^ (Table 3; Fig. 6b). Open duration histograms were fitted best with three exponential functions with open time constants O1, O2, and O3 (Fig. 6c). Unexpectedly, the L170R mutant residue opened into briefer and shorter bursts of openings, whereas the A305V mutant residue displayed openings that were similar to those observed in wt receptors. These differences in the occurrence of openings were well correlated with the single channel mean open time. Thus, L170R shortened mean open time, but A305V prolonged mean open time. However, both mutant residues reduced the open channel probability. The latter may be due to the intense desensitization that the mutant receptors possess, which would also explain the prolonged deactivation. Analysis of the relative occurrence and time constants of O1, O2, and O3 states demonstrated that L170R mainly reduced O3, while A305V mainly increased O2, states, which might have contributed significantly to the differences observed in the mean open time. The burst analysis resulted in comparable findings for wt channels in the time constant OB1 for both L170R and A305V, with a major decrease in the time constant OB2 for L170R (Table 3). However, unlike the wt receptor, receptors containing L170R or A305V had a comparable distribution in the occurrence of the two types of openings (OB1 and OB2) within bursts.

**Table 3:** Single channel properties of GABA_A_ receptors containing the β3 mutations L170R, A305V, and T288N.

	***α1β3γ2L***	***α1β3L170Rγ2***	***α1β3A305Vγ2***	***α1β3T288Nγ2***	
*Number of openings*	15128 (n = 7)	42918 (n = 11)	13853 (n = 9)	23189 (n = 6)	10177 (n = 6)
*i, pA*	2.03 ± 0.37	1.81 ± 0.20	1.52 ± 0.08***	1.59 ± 0.04**	0.93 ± 0.198
*Open probability*	0.78 ± 0.16	0.28 ± 0.14***	0.42 ± 0.34*	0.52 ± 0.26	0.42 ± 0.22*
*Mean open time, ms*	12.8 ± 3.17	4.59 ± 0.56****	17.0 ± 3.10**	6.74 ± 1.43***	11.2 ± 4.39
*O1, ms (%)*	2.21 (72)	3.68 (82)	4.61 (41)	3.71 (83)	1.96 (55)
*O2, ms (%)*	12.7 (13)	6.82 (15)	21.8 (58)	13.7 (13)	11.4 (37)
*O3, ms (%)*	59.2 (15)	14.3 (3)	13.7 (13)	44.9 (4)	67.6 (8)
*Burst duration, ms*	29.4 ± 10.5	10.5 ± 1.47****	42.2 ± 10.0**	25.3 ± 4.90	17.9 ± 5.89*
*OB1, ms (%)*	3.37 (80)	3.53 (49)	4.35 (46)	10.3 (72)	2.57 (69)
*OB2, ms (%)*	129 (20)	16.8 (51)	73.1 (54)	60.1 (28)	49.0 (31)

Values represent mean ± S.D. *^,^ **^,^ *** and ****indicate p < 0.05, p < 0.01, p < 0.001 and p < 0.0001 (one-way ANOVA with Dunnett’s multiple comparisons test) statistically different from α1β3γ2, respectively.

Taking into account that these β3 subunit mutant residues were not directly located at the GABA binding site, our results suggested that L170R and A305V distinctly and allosterically affected channel activation. It is noteworthy that the marked contrast between brief and prolonged bursts of openings seemed to be related to the perturbations that these residues caused at the coupling junction. Thus, novel H-bond networks, like those predicted for L170R (N242-G244) and A305V (R166-V305) (Fig. 5d), may explain the stabilization of different types of openings. While A305V also reduced channel conductance, structural studies showed no perturbations in the channel pore. Our results demonstrated that perturbations of residues located at the coupling junction regulate activation of the receptor, suggesting that this structural region acts as an additional coupling-gate that is in close proximity (~23 Å) to the channel gate.

To determine whether the effect of the coupling junction mutant residues L170R and A305V on the receptor is due to a loss or gain of function, we compared the ratios of receptor potency and receptor gating (supplemental Fig. 4b), properties that correlate with the average time receptors are bound with GABA and with the receptor undergoing conformational changes between open and shut states. Thus, we computed the potency ratio of EC50 of the mutant receptors/EC50 of the wild type receptors (see Table 3), and the gating ratio of the mean open time of the mutant receptors/mean open time of the wild type receptors (see Table 2). We found that the mutant residues L170R and A305V with the largest RMS deviation at the coupling junction (supplemental Fig. 4a) and augmented desensitization-deactivation coupling (prolonged deactivation and faster desensitization, see Table 1) correlated well with increased potency ratios (<1.0, blue and purple shading, supplemental Fig. 4b). Unexpectedly, despite the finding that the coupling junction mutant residues L170R and A305V increased GABA binding, gating ratios were decreased (<1.0, purple shading) or increased (>1.0, blue shading), which seems to depend on how much the mutant residue precludes the receptor from entering into different open states (Fig. 6c).

Taking into account that the potency ratios were directly correlated with the functional effect cause by the mutant residues, the resulting potency ratio is an estimate of gain or loss of function of the receptor produced by the mutant residues. Thereby the coupling junction mutant residues L170R and A305V with potency ratios <1.0 had gain of function.

### Pore β3 subunit mutant residue T288N destabilized the GABA_A_ receptor channel gate

Of the three β3 subunit mutant residues, T288N seemed to be the least severe. Despite not having an effect on surface expression of GABA_A_ receptors (Fig. 3), T288N caused a reduction of GABA-gated currents and GABA efficacy and had a substantial effect on GABA potency (Fig. 4a), which corresponded with the impaired channel gating. Unlike the coupling junction mutant residues, T288N caused perturbations that were restricted to the channel pore (Fig. 7, and supplemental Fig. 1c). Threonine 288 corresponds to the 13′ position in the M2 helix that outlines the channel pore (Fig. 7a). Highly conserved through all GABA_A_ receptor subunits, residue T288 is located just above the activation gate of the channel (L9′ position) and is one of the residues that shapes the outer mouth of the channel pore (Fig. 7b).

To determine whether T228N caused additional defects in channel gating, we studied its effects on single channel properties (Fig. 7c). In contrast to the coupling junction mutant residues, channels containing T288N opened with two distinct conductance levels: a main conductance level of ~20 pS (1.59 pA) and a low conductance level of ~12 pS (0.93 pA). Channels containing T288N opened into larger bursts of openings (~20 pS, Fig. 7c, top panel) comparable to those displayed from wt receptors (~25 pS, Fig. 6b, top panel) and with no major differences in open probability (Table 3). Although there were no significant differences among the three open time constants, a decrease in the relative occurrence of the longest open state (O3) accounted for a reduction in mean open time. Comparable distributions in the occurrence of the two types of openings (OB1 and OB2) within bursts were found without differences in the duration of bursts. These results demonstrated that receptors containing T288N retained types of openings that resembled those observed for wt receptors, but had reduced mean open time and conductance. Conversely, the lower conductance state (~12 pS) opened into briefer bursts of openings that seemed not to affect mean open time or the three open time constants, but with a major occurrence of O2. However, the open probability and burst duration were reduced, and the latter correlated with a reduction of the OB2 state within the burst (Table 3).

To define whether the effect of the pore mutant residue T288N was a loss or a gain of function, we compared the ratios of receptor potency and receptor gating (supplemental Fig. 4b). In contrast to the coupling junction mutant residues L170R and A305V, the pore mutant residue T288N with the largest RMS deviation in the M2 domain (supplemental Fig. 4a), and conserved desensitization-deactivation coupling, correlated with a decreased of both potency (>1.0) and gating (<1.0) ratios of the receptor (red shading, supplemental Fig. 4b). Since the resulting potency ratio correlated well to a gain or loss of function of the receptor, the pore mutant residue T288N had a potency ratio >1.0 and thus had a loss of function.

The presence of single channels with two different conductance states may be caused by the coexistence of αβ and αβγ heteropentameric receptors^30^. However this explanation does not apply to T288N due the lack of effect on receptor surface expression and lack of increased Zn^+2^ sensitivity. An alternative explanation is that due to its position in the extracellular entrance of the pore just above the L9′ position, T288N alters the activation gate. To gain insight into whether the T288N perturbed the open conformation of the channel pore, we determined the properties of the channel cavity and the pore-lining residues along the axis of the channel pore through the implementation of the fully-automatic method ChExVis^32^. Thus, 3D transmembrane domains of both wt and T288N receptor models in the open state were uploaded as PDB files into the ChExVis web-server using default values. The resultant transmembrane pores were extracted and visualized as skin surface (Fig. 7d, top panels) around the pore lining residues of the 3D structures (Fig. 7d). This analysis results in the 2D profile views that summarize changes in the diameter of the pore channel and hydrophilic properties within the pore (Fig. 7d, bottom panels).

Analogous to GlyRα^29^ and GluCL^19,21^ receptors, the channel pore of GABA_A_ receptors can be described as an asymmetrical hour-glass-like cavity, where the upper half is the extracellular entrance, the lower half is the intracellular entrance, and the two vestibules are constrained at the L9′ position, the activation gate. As revealed by the hydrophobicity profile, in both wt and T288N receptor structures, the intracellular entrance of the channel pore, which corresponded to lining residues at −2′ positions, were more hydrophilic than the remainder of the channel pathway. However, major differences were observed among the lining residues at positions 20′, 13′, 9′ and 6′. T288N inverted the hydrophobic pattern on both sides of the 13′ position, leaving the more cytoplasmic portion of the cavity (13′–9′) less hydrophobic and the more extracellular portion of the cavity (20′–13′) more hydrophobic than the wt structure. In addition, the L9′ radius was reduced from 5.6 Å to 5.4 Å with T288N. Interestingly it appeared that the pore pathway was elongated, while the entrance to the extracellular vestibule was shortened at the activation gate. This latter may be due to the presence of an unusual histidine lining the channel pore at the extracellular entrance of the structure with T288N (Fig. 7b). These observations support the theory that the protrusion of T288N into the pore channel leads to perturbations of the flow of chlorine ions through the pore. This results in different conductance levels in the channel open state.

## Discussion

Our data provide new directions for understanding the molecular mechanisms governing dysfunction of GABA_A_ receptors in patients with epileptic encephalopathies. To date there have been more than 46 reported *de novo* missense mutations associated with severe cases of EOEE^6–10,33–39^ in GABA_A_ receptor genes commonly associated with haploinsufficiency and hyperexcitability. Remarkably, the distributions of these mutant residues throughout the α1, β(1, 2, 3), and γ2L subunits are quite comparable. Of all reported mutant residues, 33% were in α1, 52% were in β(1, 2, 3), and 15% were in γ2L subunits. Comparing the occurrence of these mutant residues by structural domains, they occur more frequently in the transmembrane (61%) domain than in the N-terminal (39%) domain of the receptor. Those found in the N-terminal domain were located close to the GABA-binding interface^6,9,10,34,36,39^. This is in contrast to what was reported for β3 and γ2L mutant residues located in the uppermost region of the N-terminal domain, which produce mild epilepsy^40–43^. Although mutant residues were widespread among subunits, there are common features that suggest an association between structure and function. As part of the pentameric ligand-gated ion channel (LGIC) family, GABA_A_ receptor subunits share the same architectural core structure; whereby each subunit contains conserved “structural cassettes” that lead to activation of the different receptors in a similar way. Shedding light on whether or not mutant residues linked to EE cause rearrangements in these conserved “structural cassettes” may clarify the molecular basis for common disorders in different genes and highlight the epileptogenic nature of these structures.

A total of 23 likely pathogenic *de novo* missense mutations associated with EOEE were identified in genes encoding the GABA_A_ receptor β1^6,37^, β2^38,39^, and β3^6–10^ subunits (supplemental Fig. 2). Mapping these mutant residues on the β subunit clearly demonstrates that they occur predominantly within the β( + )/α(−) interfaces along the pore axis that is directly related to receptor activation. We studied three β3 subunit missense mutant residues, L170R^8^, T288N (novel), and A305V^8^. These residues occur in spatially separated regions of the secondary structure of the receptor, but share localization in two “structural cassettes” that are directly related to the transduction of the binding of GABA in the N-terminal domain to the activation of the channel with the opening of the pore. These residues are located at the intersection between the N-terminal domain and the channel pore in the coupling junction of the receptor. Whereas the β3 subunit coupling junction mutant residues L170R and A305V uncoupled during activation and caused gain of function, the β3 subunit pore mutant residue T288N favoured low conductance receptors and caused loss of function. Furthermore, only the coupling junction mutant subunits impaired (to a minor extent) the trafficking of the mutant subunits to the surface, which was expected since homologous assembly motifs are embedded in the same region^14,15^. It is noteworthy that homologous missense mutations at A305 and T288 residues have also been reported. A *de novo* β3 subunit mutant residue A305T was identified in a patient with Lennox-Gastaut syndrome^10^, while the β2 subunit mutant residue T287P, which shares the homologous position of the β3 subunit pore mutant residue T288N, was described in a case of myoclonic atonic epilepsy^38^. This is a distinctive difference between missense mutant residues associated with moderate epilepsies, which are more prone to cause trafficking/assembling impairment and ER retention of the unfolded mutants^44–48^. Thus, it appears that missense mutant residues in “structural cassettes” of the receptor that are not directly coupled to activation tend to cause more trafficking defects, than those in domains coupled directly to the activation of the channel, and so the kinetic defects predominate^11,49^. This is an interesting concept because in mild epilepsies associated with missense mutant residues the main mechanism shaping the final phenotype appears to be the result of secondary effects caused by deficient protein quality control of unassembled mutant subunits. Severe epilepsies associated with missense mutant residues are caused directly by the ongoing present of dysfunctional mutant subunits on the cell surface, not a lack of them. It seems for patients with epilepsy to have receptors functioning abnormally (i.e. mutant receptors) is worse than maintaining a smaller number of receptors that work well (i.e. wild type receptors), which may in turn determine the difference between mild and severe epilepsies.

Our data demonstrated that mutant residues that occurred at the coupling junction impaired a “structural cassette” that modulated the gating efficiency of the receptor. It is well known that during activation of LGICs^19,21,29^, conformational changes in subunits that are initiated in the N-terminal domain are transmitted as much as 50 Å away through loops and β-strands to reach the activation gate in the channel pore. However it is not clear what the role of the junction between these two domains is during channel activation. In line with our results, a previous study described that clusters of residues within the core of the protein between the Cys-loop, β1-β2 loop and the M2-M3 linker stabilized the open state of the receptor^50^. Most striking is that the residues perturbed within the four nearby regions at the interface by L170R and A305V are among the most conserved residues of the LGIC family^51^. Indeed, L170 is part of the highly conserved F/YPxD motif at the tip of the Cys-loop, where the first residue corresponds to Y168 and ‘x’ to the L170 position in the β3 subunit. Another residue with conservation above 80% is R241 in the pre-M1 region, which was also perturbed in our studies. Thus, it is expected that the occurrence of mutant residues in this region, such as those reported in patients with *de novo* missense mutant β3(S76C)^9^, β2(M79T)^39^, β1(F246S)^6^, β3(A305T)^10^, β3(L293H)^10^ and β3(Y302C)^6,9^ subunits (supplemental Fig. 2), alter this “structural cassette” and cause a similar phenotypic range of EOEE. In a second “structural cassette”, outlining the transmembrane M2 α helix in the channel pore, the pore radius at L9′ was reduced by the pore mutant residue T288N. Even though the primary sequence of residues in the transmembrane domains are the least conserved among the LGIC family^51^, the L9′ position in M2 has a conservation above 75%. This is in consistent with the high structural conservation of the α-helices of the four transmembrane segments (M1 to M4) that delineate the channel pore^19,21,29^. Consequently, the structural conservation of the pore domain predicts similar perturbations caused by concurrent missense mutant residues, and thus similar epileptic syndromes^7,9,10,37,38^ (supplemental Fig. 1). Our results highlight the importance of these highly conserved “structural cassettes” throughout the LGIC family and confirms the evolutionary accumulation of missense mutant residues in homologous regions and in different receptor subunits that result in similar hyperexcitability phenotypes^52–58^.

## Materials and Methods

All experimental procedures were performed in accordance with the National Institutes of Health Guide for the Care Policies, Procedures and Regulations and were approved by the Vanderbilt University Medical Center Safety/Environment Committee to ensure safe practices. Parental/guardian consent was obtained for the use and publication of sensitive proband images and submitted for approval according to the Ethics Review Committee of the Peking University First Hospital Medical. Parents of each proband provided signed informed consent using a protocol approved by the Ethics Review Committee of the Peking University First Hospital Medical.

### Patient phenotypes

Three male patients were selected for sequencing because they had recurrent seizures and severe cognitive and motor development impairment with early infantile onset. Genomic DNA was extracted from peripheral leukocytes from three trios with no epilepsy or any related history for segregation analysis^8^. Written informed consent was obtained from the probands. Table S1 gives details of the probands. This study was approved by the Peking University First Hospital Medical Ethics Committee.

### Targeted next-generation sequencing and analysis

Custom-designed panels capturing the coding exons of *GABRB3* were synthetized using the Agilent SureSelect Target Enrichment technique. Targeted next-generation sequencing was subsequently performed on an Illumina GAIIx platform (Illumina, San Diego, CA, U.S.A.) using a paired-end sequencing of 110 bp to screen for mutations as described previously. We used Sanger sequencing to confirm the origin of the mutation as being *de novo*. We used a custom-designed gene panel from a total of 480 candidate genes associated with epilepsy and IDDS (Table S2), including their exons and exon-intron boundaries (1.285 M bp in total).

### cDNA constructs and expression of recombinant GABA_A_ receptors

cDNAs encoding human GABA_A_ receptor α1 (NM_000806.5), β3 (NM_021912.4 variant 2) and γ2L (NM_198904.2) subunits and EGFP (LC008490.1) were cloned into the pcDNA(3.1+) vector. The three mutations in the cDNA encoding the β3 subunit were introduced using the QuikChange Site-Directed Mutagenesis kit (Agilent). HEK293T cells (HEK 293 T/17, ATCC^®^ CRL-11268^™^) were cultured as monolayers at 37 °C in Dulbecco’s Modified Eagle Medium (Invitrogen) supplemented with 10% fetal bovine serum (Invitrogen) and 100 IU/ml each of penicillin and streptomycin (Invitrogen). For surface biotinylation and flow cytometry experiments, cells were plated at a density of 4–6 × 10^5^ in 60 mm culture dish (Corning) and transfected 24 hours after plating. For electrophysiology experiments, cells were plated at 4 × 10^4^ in 35 mm culture dishes and transfected after 24 hours with 0.3 μg cDNA of each α1, β3, and γ2L subunits and 0.05 µg of EGFP using X-tremeGENE9 DNA Transfection Reagent (Roche Diagnostics).

### Surface biotinylation and western blot

The surface and total expression levels of wild type α1, β3, γ2L and mutant β3(L170R), β3(A305V) and β3(T288N) subunits were determined as described previously. Primary antibodies against human α1 subunits (N-terminal, clone BD24, Millipore; 2.5 g/ml), human β3 subunits (N-terminal, monoclonal, β2/3-PE, clone 62–3G1, Millipore; 2.5 g/ml), human γ2L subunits (clone S96-55, Novus Biologicals) and the HA epitope tag (clone 16B12, Covance; 2.5 g/ml) were used to detect GABA_A_ receptor subunits. For western blot experiments, Na^+^/K^+^-ATPase protein was used as a loading control (0.2 g/ml, clone 464.6, ab7671, Abcam), and anti-mouse IRdye conjugated secondary antibodies (Li-Cor) were used in all cases. Membranes were scanned using the Li-Cor Odyssey system, and integrated intensities of bands were determined using Odyssey software.

### Immunocytochemistry and confocal microscopy

Transfected HEK293T cells were fixed with Prefer (Anatech) to stain surface proteins. The fixed cells were then stained with mouse monoclonal β2/3 subunit antibody (Millipore) and rabbit monoclonal HA antibody (Cell Signalling) overnight, followed by incubation in Cy3-conjugated donkey anti-mouse IgG antibodies and Alexa 488-conjugated donkey anti-rabbit IgG antibodies. Coverslips were mounted with Prolong Gold antifade reagent (Thermo Fisher Scientific Inc.). Confocal images were obtained from HEK293T cells using a Zeiss LSM 710 Meta inverted confocal microscope. Stained HEK293T cells were excited with the 543 nm laser for the Cy3 fluorophore signal and the 488 nm laser for the Alexa 488 fluorophore signal. Images were taken with 8 bit, 1024 × 1024 pixel resolution. Pinholes were adjusted so that the sample thickness was 0.9 μm. An average of four scans was taken to decrease the background noise. Confocal experiments were performed in part through the use of the VUMC Cell Imaging Shared Resource.

### Electrophysiology

Whole cell recordings from lifted HEK293T cells and cell attached single channel recordings were obtained as previously described^11,59^. For whole cell recordings the external solution was composed of (in mM): 142 NaCl, 8 KCl, 10 D( + )-glucose, 10 HEPES, 6 MgCl_2_.6H_2_O, and 1 CaCl_2_ (pH 7.4, ~326 mOsm). The internal solution consisted of (in mM): 153 KCl, 10 HEPES, 5 EGTA, 2 Mg-ATP, and 1 MgCl_2_.6H_2_O (pH 7.3, ~300 mOsm). This combination of external and internal solutions produced a chloride equilibrium potential of ~0 mV, and cells were voltage clamped at −20 mV. Drugs were gravity-fed to four-barrel square glass connected to a SF-77B Perfusion Fast-Step system (Warner Instruments Corporations). The solution exchange time across the open electrode tip was ~200–400 μs, and the exchange around lifted cells (~8–12 pF) occurred within 800 μs, which was sufficiently fast for these experiments^16^ and guaranteed rapid solution exchanges and accurate measurement of the kinetic properties of the receptor. All experiments were performed at room temperature (22–23 °C). Single-channel currents were recorded in an external solution containing (in mM): 140 NaCl, 5 KCl, 1 MgCl_2_, 2 CaCl_2_, 10 glucose, and 10 HEPES (pH 7.4). During recording, 1 mM GABA was present in the internal solution consisted of (in mM): 120 NaCl, 5 KCl, 10 MgCl_2_, 0.1 CaCl_2_, 10 glucose, 10 HEPES, and 1 mM of GABA (pH 7.4). The micropipette potential was + 80 mV.

Whole cell and single channel currents were amplified and low-pass filtered at 2 kHz using an Axopatch 200B amplifier, digitized at 10 kHz (whole cell recordings) or 20 kHz (single channel recordings) using Digidata 1550, and saved using pCLAMP 10.4 (Axon Instruments). Data were analysed offline using Clampfit 10.4 (Axon Instruments, TAC 4.2 and TACFit 4.2 (Bruxton Corporation) software. Activation and deactivation current time constants (τ) were measured by application of 1 mM GABA for 10 ms, while desensitization and peak current amplitude were measured by application of 1 mM GABA for 4 s. Activation, desensitization and deactivation current time courses were fitted using the Levenberg–Marquardt least squares method with up to four component exponential functions of the form ∑*a*_*n*_exp(−*t*/τ_*n*_) + *C*, where *n* is the number of the exponential components, *t* is time, *a* is the relative amplitude, τ_*n*_ is the time constant, and *C* is the residual current. Additional components were accepted only if they significantly improved the fit, as determined by an *F* test on the sum of squared residuals. The time course of deactivation was summarized as a weighted time constant, defined by the following expression: ∑*a*_*n*_τ_*n*_/∑*a*_*n*_. The extent of desensitization was measured as (fitted peak current–fitted steady-state current)/(fitted peak current). GABA_A_ receptor current concentration–response curves were fitted using GraphPad Prism version 6.07 for Windows (GraphPad Software, La Jolla, CA). We used a nonlinear regression Hill equation of the form E = E_basal_ + (E_MAX_ − E_basal_)/(1 + 10((LogEC_50_ − X) * Hill Slope)), where E is the fractional response of the GABA-gated currents, E_max_ is the maximal response, x is [GABA], EC_50_ is the [GABA] at which response = 50% of maximal response, and *nH* is the Hill coefficient. Thus, the *nH* for wt and L170R, A305V, and T288N GABA_A_ was 1.09 ± 0.17, 3.47 ± 0.71, 0.40 ± 0.26, and 2.28 ± 0.28, respectively. Potentiation of GABA-gated currents by 1 µM diazepam or 1 µM diazepam + 10 µM Zn^2+^ was measured by coapplication with 1 µM GABA for 4 s. Peak GABA-gated current amplitudes measured before and after the compound coapplication were compared to determine the ability of diazepam to augment GABA-gated currents. Inhibition of 1 mM GABA-gated currents by 10 µM Zn^2+^ was measured by pre-application for 10 s followed by coapplication with GABA for 4 s. GABA, diazepam and Zn^2+^ were obtained from Sigma.

Single-channel open and closed events were analysed using the 50% threshold detection method and visually inspected before accepting the events. Single-channel openings occurred as bursts of one or more openings or clusters of bursts. Bursts were defined as one or more consecutive openings that were separated by closed times that were shorter than a specified critical duration (*t*_crit_) prior to and following the openings^60^. A *t*_crit_ duration of 5 ms was used in the current study. Clusters were defined as a series of bursts preceded and followed by closed intervals longer than a specific critical duration (*t*_cluster_). A *t*_cluster_ of 10 ms was used in this study. Open and closed time histograms as well as amplitude histograms were generated using TACFit 4.2 (Bruxton Corporation, Seattle, WA, USA). Single-channel amplitudes (*i*) were calculated by fitting all-point histograms with single- or multi-Gaussian curves. The difference between the fitted ‘closed’ and ‘open’ peaks was taken as *i*. Duration histograms were fitted with exponential components in the form: ∑(*a*_*i*_ */*τ_*i*_)exp(−*t*/τ_*i*_), where *a* and τ were the relative area and the time constant of the *i* component, respectively, and *t* as the time. The mean open time was then calculated as follows: ∑*a*_*i*_τ_*i*_. The number of components required to fit the duration histograms was increased until an additional component did not significantly improve the fit^30^.

### Structural modelling and simulation

3D structural models of the GABA_A_ receptor in the open, closed and desensitized conformation states were simulated using the electron cryo-microscopy of the *Danio rerio* glycine receptor α1 subunit (GlyRα) structures^29^ in the open (3JAE), closed (3JAD) and desensitized (3JAF) conformation states as templates. GABA_A_ receptor α1, β3 and γ2 subunit raw sequences in FASTA format were individually loaded into Swiss-PdbViewer 4.10^61^ for template searching against ExPDB database (ExPASy, http://www.expasy.org/). The initial sequence alignments between GABA_A_ receptor α1, β3 and γ2 subunits and *Danio rerio* GlyRα subunits in the open (3JAE), closed (3JAD) and desensitized (3JAF) conformation states were generated with full-length multiple alignments using ClustalW. Sequence alignments were inspected manually to assure accuracy among structural domains solved from the template. Because the quite long M3/M4 cytoplasmic loop of the GABA_A_ receptor subunits was absent in the solved glycine receptor structures, the correspondent fourth transmembrane domains (M4) were misalignment onto the template. Consequently, the M3/M4 cytoplasmic loop was excluded from the modelling, and separate alignments were generated for the M4 domains. Then full-length multiple alignments were submitted for automated comparative protein modelling implemented in the program suite incorporated in SWISS-MODEL (http://swissmodel.expasy.org/SWISS-MODEL.html). Before energy minimization, resulting 3D models of human GABA_A_ receptor α1, β3 and γ2 subunits in the three conformational states were inspected manually, their structural alignments confirmed, and evaluated for proper h-bonds, presence of clashes and missing atoms using Molegro Molecular Viewer (www.clcbio.com). Then, pentameric 3D GABA_A_ receptor models were generated by combining α1, β3 and γ2 structural models in the stoichiometry 2β:2α:1γ with the subunit arrangement β3-α1-β3-α1-γ2L in a counter-clockwise order by superposition onto the *Danio rerio* GlyRα in the open (3JAE), closed (3JAD) and desensitized (3JAF) conformational states. Neighbourhood structural conformational changes within a radius of 6 Å of the mutated residue in the β3 subunit in the 3D structural models of the GABA_A_ receptor in the open, closed and desensitized conformation states were simulated using Rosetta 3.1^62^, implemented in the program suite incorporate in Rosetta Backrub (https://kortemmelab.ucsf.edu). Up to twenty of the best-scoring structures were generated at each time by choosing parameters recommended by the application. RMS deviations were calculated between the initial (wild type) structures and superimposes simulated (mutated) structures. For each 3D GABA_A_ receptor conformation state, the RMS average over ten low energy structures was computed and conformational changes displayed among neighbourhood structural domains. The molecular channel extraction and visualization of the 3D structural models of the wt and the pore mutation T288N in the open conformation state were determined using ChExVis^32^, a computational automated method available as a web-based resource (http://vgl.serc.iisc.ernet.in/chexvis/). From the ChExVis outputs, a list of the identified pore-lining residues of the transmembrane extracted pore, and the given pore diameter profile and hydrophobicity of the structures were used. The models were rendered using USF Chimera version 1.10^63^.

### Statistical Analysis

Numerical data were expressed as mean ± S.E.M or S.D as indicated. Statistical analysis was performed using GraphPad Prism (GraphPad Software 6.07). Statistical significance was taken as *p* < 0.05, using unpaired two-tailed Student’s t test and one-way ANOVA with Dunnett’s multiple comparisons test as appropriate.

## Electronic supplementary material

SREP-17-19725A-Supplemental information
