# Supplementary material for: GABAA Receptor Coupling Junction and Pore GABRB3 Mutations are Linked to Early-Onset Epileptic Encephalopathy
Source: Sci Rep. 2017 Nov 21;7:15903. doi: 10.1038/s41598-017-16010-3 (PMC5698489; doi:10.1038/s41598-017-16010-3)
Supplement: Supplementary file 1 — SREP-17-19725A-Supplemental information [file 41598_2017_16010_MOESM1_ESM.pdf]

# GABA$_A$ Receptor Coupling Junction and Pore *GABRB3* Mutations are Linked to Early-Onset Epileptic Encephalopathy

Ciria C. Hernandez*[1], Yujia Zhang[3], Ningning Hu[1],

Dingding Shen[2], Wangzhen Shen[1], Xiaoyan Liu[3], Weijing Kong[3], Yuwu Jiang*[3] and Robert L. Macdonald*[1]

[1]Department of Neurology, Vanderbilt University, Nashville, TN. USA.

[2]The Graduate Program of Neuroscience, Vanderbilt University, Nashville, TN.

[3]Department of Pediatrics, Peking University First Hospital. Beijing, China.

## Supplementary information

***Supplemental Figure 1***

***Supplemental Figure 1: Network of adjacent interactions among residues that were located in bordering regions at the coupling junction.***

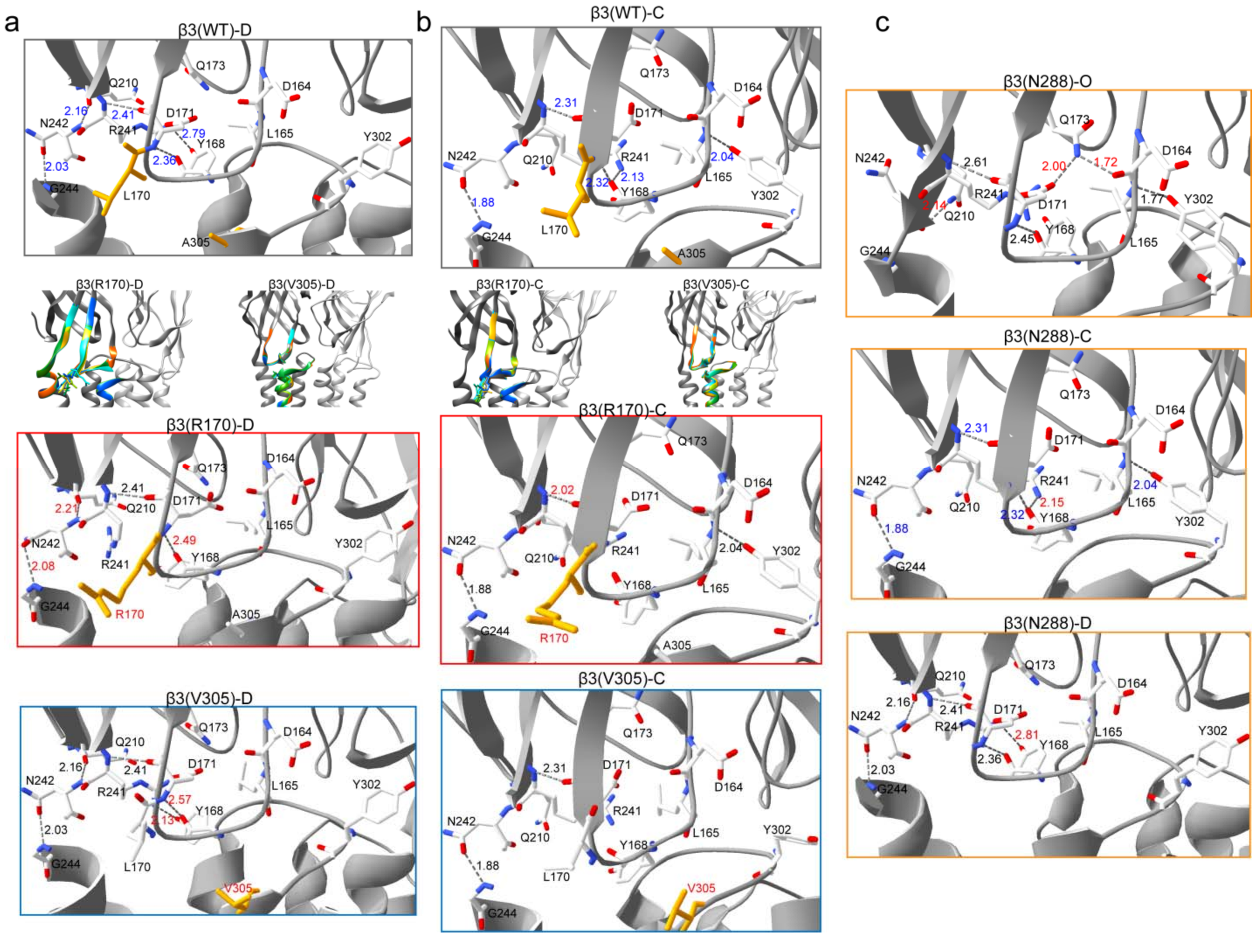


The predicted H-bonds for the L170R, A305V, and T288N models in the three states of the receptor were shown. The four domains and their amino acids were represented as in Figure 5. C, closed state. O, open state. D, desensitized state.

***Supplemental Figure 2***

***Supplemental Figure 2: Mapping of EOEE associated GABRB de novo mutations in the GABA$_A$ receptor***

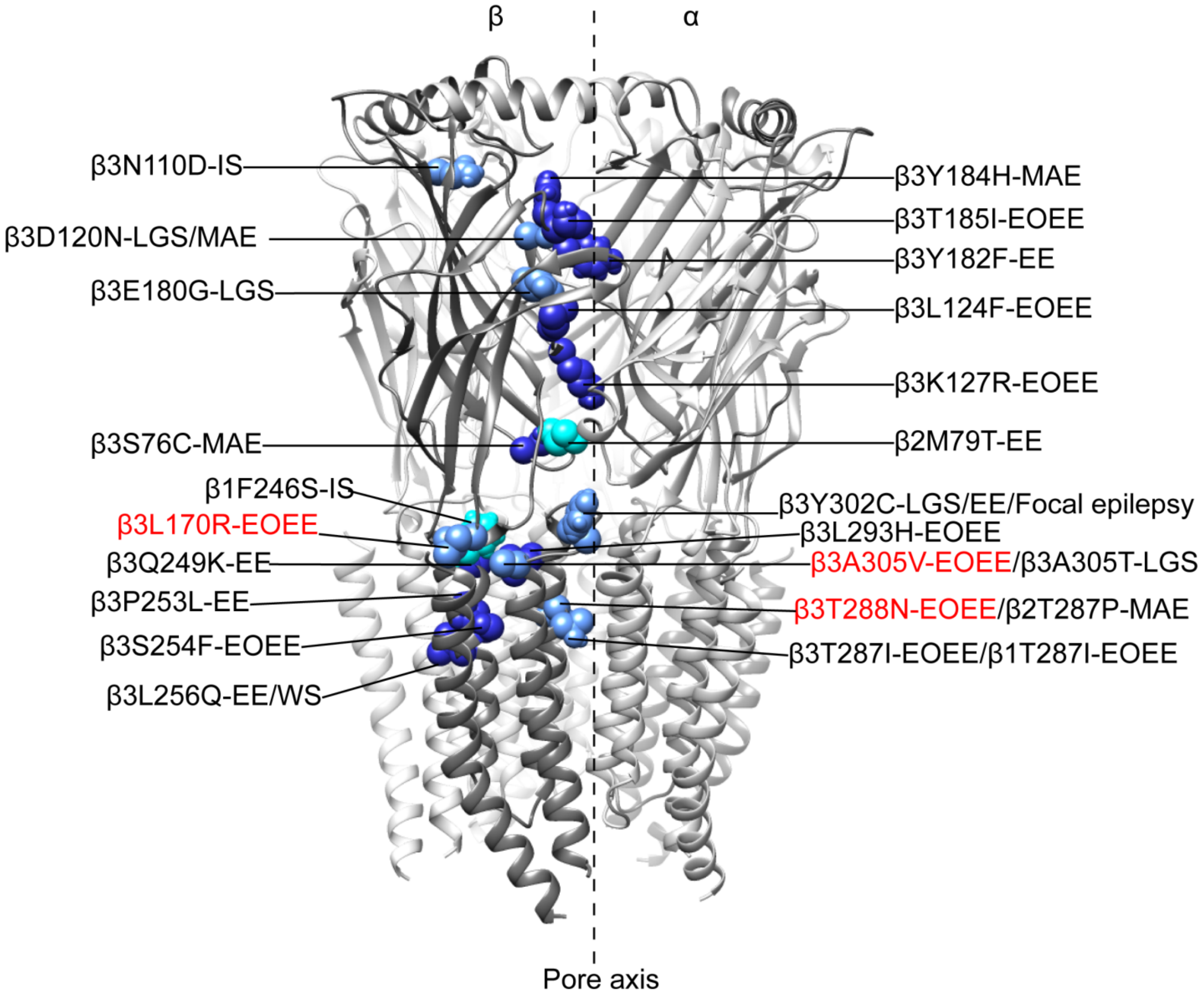


3-D structural model of the pentameric GABA$_A$ receptor structure at the β/α subunit interface mapping the *GABRB* mutations. The residues are shown is space filled representation and in different shades of blue for clarity (see details in the text).

***Supplemental Figure 3***

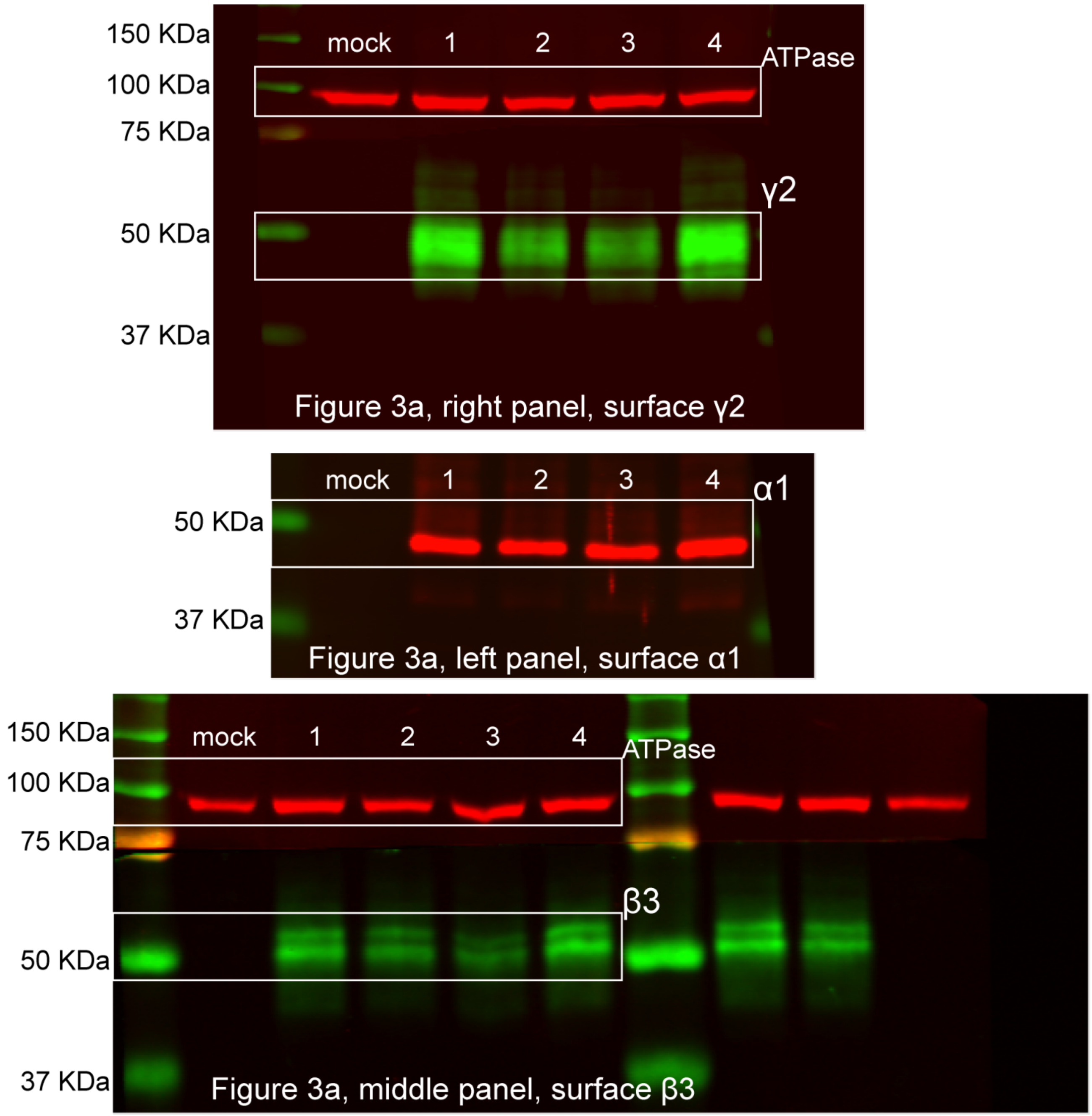


***Supplemental Figure 3:*** Full-length gels for images shown in Figure 3a. The boxed region in each gel represents the cropped image shown in Figure 3a for each panel, respectively. Note that the loading control (ATPase) was the same for γ2 and α1 surface expression.

***Supplemental Figure 4***

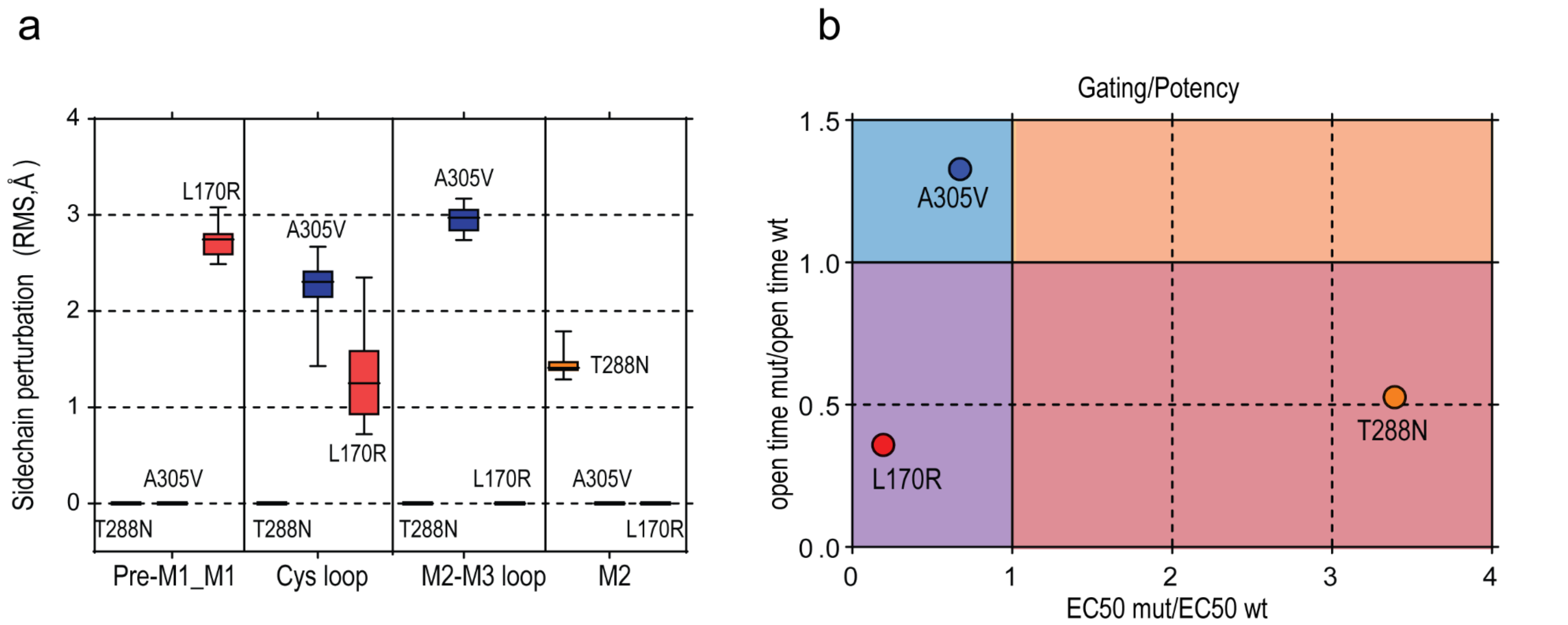


***Supplemental Figure 4:*** (**a**), box plots show predicted perturbations caused by the mutant sidechain residues L170R, A305V and T288N at Pre-M1_M1 (R241-Y245), Cys-loop (L165-Y168), M2-M3 loop (P298-Y302), and M2 (T281-E295) domains in the open conformation. RMS values for up to 10 simulations are represented as interleaved box and whiskers (25-75% percentile, median, and minimum and maximum). (**b**), Correlation of the potency and gating ratio plots for the coupling junction mutant residues L170R and A305V, and the pore mutant residue T288N. Ratios equal to 1 indicates no changes when compared to wild type (wt).

***Table S1. Clinical Features in 3 Patients With EOEE and GABRB3 Missense Mutations.***

| Case (Sex) | Age | Age at seizure onset | Diagnosis | Seizure types | Neurodevelopment delay and Cognitive outcome | EEG Finding | Brain imaging | Response to AED |
|---|---|---|---|---|---|---|---|---|
| PED 1, proband II-3 (M) | 3 y | 6 m | EOEE | Focal and secondary generalized tonic–clonic seizures (GTCS) | Severe delay at motor and language--delayed developmental milestones, and psychomotor regression, hypotonia and the rest was unremarkable. Poor coordination. | Generalized fast-wave in background activity; a large amount of widespread medium to high volatility multifocal sharp and spike discharges in anterior region during sleep (at 1 year). | Thin corpus callosum | Refractory to AEDs |
| PED 2, proband II-3 (M) | 9 y | 6 m | EOEE | Focal and secondary generalized tonic–clonic seizures (GTCS) | Severe delay at motor and language, unable to lift and hold the head upright or to sit, unable to recognize people and nonverbal at 1 year old. Limited comprehension. Very limited progression when seizures were overcome. Poor coordination. | Generalized multifocal low to high volatility sharp, spike, sharp-slow and spike-slow wave complexes in the left occipital and the posterior of temporal region, significant increased during sleep period, spread gradually to the opposite sides of same regions and same side of anterior regions (at 1 year). | Mild cortical atrophy (mainly frontal lobe). | Refractory to AEDs |
| PED 3, proband II-3 (M) | 1 y 6 m (death) | 3 m | EOEE | Partial seizures. Eyelid myoclonus with stiffening hands, or companied with eye deviation. The seizure onset usually lasted 10 sec. to 3 min., multiple times per day. | Severe delay at motor and language--unable to lift and hold his head upright or roll over at 6 months age, and unable to understand or express language at over 1 year old. Hypotonia and the rest were unremarkable. Poor coordination. | Bilateral discharges during inter-ictal period and focal spike wave discharges in the right temporal region during ictal period (at 5 months). | Mild cortical atrophy (mainly frontal lobe). | Refractory to AEDs |

PED, pedigree; M, male; y, years; m, moths; EOEE, early onset epileptic encephalopathy; AEDs, antiepileptic drugs.

***Table S2. A list of 480 Candidate Genes Associated with Epileptic Encephalopathies***

| Gene Symbols | | | | | | | | | | | | | |
|---|---|---|---|---|---|---|---|---|---|---|---|---|---|
| ABAT | ASS1 | CHRFAM7A | DDC | GABRA4 | GPR98 | IL6ST | KCNK1 | MAP2 | PAFAH1B1 | PNKD | SCN2A | SLC6A13 | TPP1 |
| ABCC2 | ATIC | CHRNA2 | DEPDC5 | GABRA5 | GRIA1 | IMPA2 | KCNK3 | MBD5 | PAH | PNKP | SCN2B | SLC6A19 | TPRXL |
| ABCC8 | ATN1 | CHRNA4 | DGKD | GABRA6 | GRIA2 | INA | KCNK7 | MBTPS2 | PALLD | PNPO | SCN3A | SLC6A8 | TREX1 |
| ACADM | ATP1A2 | CHRNA5 | DHFR | GABRB1 | GRIA3 | IQSEC2 | KCNK9 | ME2 | PARK2 | POLG | SCN3B | SLC9A3 | TRMT44 |
| ACO2 | ATP1A3 | CHRNA7 | DIAPH3 | GABRB2 | GRIA4 | JRK | KCNMA1 | MECP2 | PC | POLG2 | SCN4A | SLC9A6 | TRPM6 |
| ACOX1 | ATP2A2 | CHRNB2 | DLX2 | GABRB3 | GRIK1 | JUN | KCNMB2 | MED17 | PCDH19 | PPP1R3C | SCN5A | SLC9A9 | TSC1 |
| ACP1 | ATP6AP2 | CLCN2 | DLX5 | GABRD | GRIK2 | KARS | KCNMB3 | MEF2C | PDCD10 | PPT1 | SCN7A | SMARCA2 | TSC2 |
| ACSF3 | ATP6V0A2 | CLCN4 | DNM1 | GABRE | GRIN1 | KCNA1 | KCNMB4 | MFSD8 | PDHA1 | PRF1 | SCN8A | SMARCA4 | TSEN2 |
| ACTA2 | ATP7A | CLCNKB | DPM1 | GABRG1 | GRIN2A | KCNA2 | KCNN2 | MIB1 | PDHX | PRICKLE1 | SCN9A | SNIP1 | TSEN34 |
| ACTB | AUH | CLEC18A | DPYS | GABRG2 | GRIN2B | KCNA6 | KCNQ1 | MMADHC | PDYN | PRICKLE2 | SDHA | SOBP | TSEN54 |
| ACY1 | BCKDK | CLIC2 | DYRK1A | GABRG3 | GRIN2C | KCNAB1 | KCNQ2 | MOCS1 | PEX1 | PRNP | SEPSECS | SPAST | TSPO |
| ADAR | BDNF | CLN3 | EFHC1 | GABRP | GRIN2D | KCNAB2 | KCNQ3 | MOCS2 | PEX10 | PRODH | SERPINI1 | SPR | TUBA1A |
| ADCK3 | BRAT1 | CLN5 | EFHC2 | GABRQ | GRIN3A | KCNAB3 | KCNQ4 | MOG | PEX12 | PRRT2 | SEZ6 | SPTAN1 | TUBA8 |
| ADK | BRD2 | CLN6 | EGF | GABRR1 | GRIN3B | KCNB1 | KCNQ5 | MSN | PEX13 | PSAP | SHANK3 | SRGAP2 | TUBB2B |
| ADSL | BTD | CLN8 | EHMT1 | GABRR2 | GRM1 | KCNC1 | KCNS2 | MTHFR | PEX14 | PSAT1 | SLC12A1 | SRPX2 | TUBGCP6 |
| AFG3L2 | C10ORF2 | CNP | EIF2AK3 | GABRR3 | GRN | KCNC2 | KCNS3 | MTMR9 | PEX16 | PSEN1 | SLC12A5 | ST3GAL3 | U2AF1 |
| AGTR2 | CA1 | CNR1 | ELOVL4 | GAD1 | HAX1 | KCNC4 | KCNT1 | MTR | PEX19 | PTEN | SLC12A6 | ST3GAL5 | UBC |
| ALDH4A1 | CACNA1A | CNTF | ELP4 | GAMT | HCCS | KCND1 | KCNV1 | NAGA | PEX2 | PTH | SLC16A1 | STRADA | UBE2A |
| ALDH5A1 | CACNA1E | CNTN2 | EMX2 | GATM | HCFC1 | KCND2 | KCNV2 | NDE1 | PEX26 | PTS | SLC17A5 | STXBP1 | UBE3A |
| ALDH7A1 | CACNA1G | CNTNAP2 | EN2 | GBA | HCN1 | KCNE1L | KCTD7 | NDP | PEX3 | QDPR | SLC19A3 | SUOX | VAMP2 |
| ALG13 | CACNA1H | COG6 | EPM2A | GCDH | HCN2 | KCNE2 | KDM5C | NDUFV1 | PEX5 | RAB39B | SLC1A1 | SV2A | VLDLR |
| AMACR | CACNA2D2 | COG8 | EPM2AIP1 | GCH1 | HCN4 | KCNF1 | KLK1 | NF1 | PEX6 | RANBP2 | SLC1A2 | SYN1 | VPS13A |
| AMT | CACNG2 | COL4A1 | FADD | GCM2 | HDAC4 | KCNG1 | KRIT1 | NGLY1 | PFKL | RBFOX1 | SLC1A3 | SYNGAP1 | WARS |
| ANK3 | CALHM1 | COQ2 | FARS2 | GCSH | HEPACAM | KCNG4 | L1CAM | NHLRC1 | PFKM | RBPJ | SLC20A2 | SYP | WDR45 |
| ANKRD11 | CASK | COQ9 | FKTN | GJA1 | HEXA | KCNH2 | LAMA2 | NIPA2 | PHF6 | RELN | SLC25A12 | SYT11 | ZEB2 |
| AP4E1 | CASP9 | CPA6 | FLNA | GJD2 | HEXB | KCNH3 | LAMB1 | NPY | PHGDH | RHAG | SLC25A13 | SZT2 | |
| ARG1 | CASR | CPS1 | FOLR1 | GLB1 | HLCS | KCNH8 | LBR | NRXN1 | PHOX2A | RHOA | SLC25A22 | TBC1D24 | |
| ARHGEF15 | CBS | CPT2 | FOS | GLDC | HNRNPU | KCNJ1 | LGI1 | NSDHL | PIGA | RMND1 | SLC2A1 | TBCE | |
| ARHGEF9 | CCDC88C | CSTB | FOXG1 | GLRA1 | HOXA1 | KCNJ10 | LGI2 | NSF | PIGL | RNASEH2A | SLC35A2 | TBX1 | |
| ARSA | CCL3 | CTDP1 | FUCA1 | GLRB | HPD | KCNJ11 | LGI4 | NTRK1 | PIGN | RNF213 | SLC35A3 | TCF4 | |
| ARX | CCL4 | CTSD | GABBR1 | GLUD1 | HSD17B10 | KCNJ2 | LIAS | NTRK2 | PIGO | ROGDI | SLC35C1 | TICAM1 | |
| ASAH1 | CCM2 | CUL4B | GABBR2 | GM2A | HTR1A | KCNJ3 | LIFR | OPA1 | PIGV | RPS6KA3 | SLC46A1 | TK2 | |

| | | | | | | | | | | | | | |
|---|---|---|---|---|---|---|---|---|---|---|---|---|---|
| ASIC1 | CDK5 | CYP4F11 | GABRA1 | GOSR2 | IDH2 | KCNJ5 | LMBRD1 | OPHN1 | PIR | SCARB2 | SLC4A3 | TLR3 | |
| ASPA | CDKL5 | D2HGDH | GABRA2 | GPHN | IDS | KCNJ6 | MANBA | OPRM1 | PKHD1 | SCN1A | SLC52A2 | TMEM67 | |
| ASPM | CHD2 | DCX | GABRA3 | GPR56 | IER3IP1 | KCNJ9 | MAOB | OTX2 | PLCB1 | SCN1B | SLC6A11 | TNF | |
